# A Comprehensive Safety Trial of Chimeric Antibody 14.18 With GM-CSF, IL-2, and Isotretinoin in High-Risk Neuroblastoma Patients Following Myeloablative Therapy: Children’s Oncology Group Study ANBL0931

**DOI:** 10.3389/fimmu.2018.01355

**Published:** 2018-06-18

**Authors:** M. Fevzi Ozkaynak, Andrew L. Gilman, Wendy B. London, Arlene Naranjo, Mitchell B. Diccianni, Sheena C. Tenney, Malcolm Smith, Karen S. Messer, Robert Seeger, C. Patrick Reynolds, L. Mary Smith, Barry L. Shulkin, Marguerite Parisi, John M. Maris, Julie R. Park, Paul M. Sondel, Alice L. Yu

**Affiliations:** ^1^New York Medical College, Valhalla, NY, United States; ^2^Levine Children’s Hospital, Charlotte, NC, United States; ^3^Dana Farber Cancer Institute and Boston Children’s Hospital, Harvard Medical School, Boston, MA, United States; ^4^Children’s Oncology Group Statistics and Data Center, University of Florida, Gainesville, FL, United States; ^5^Moores Cancer Center, University of California, San Diego, La Jolla, CA, United States; ^6^National Cancer Institute, Bethesda, MD, United States; ^7^Children’s Hospital Los Angeles, University Southern California, Los Angeles, Los Angeles, CA, United States; ^8^Texas Tech University Health Sciences Center, Lubbock, TX, United States; ^9^United Therapeutics, Silver Spring, MD, United States; ^10^St. Jude’s Children’s Research Hospital, Memphis, TN, United States; ^11^Seattle Children’s Hospital, University of Washington School of Medicine, Seattle, WA, United States; ^12^Children’s Hospital of Philadelphia, Philadelphia, PA, United States; ^13^University of Wisconsin Carbone Cancer Center, Madison, WI, United States; ^14^Institute of Stem Cell and Translational Cancer Research, Chang Gung Memorial Hospital, Taoyuan, Taiwan

**Keywords:** neuroblastoma, immunotherapy, anti-GD2 chimeric antibody, cytokines, safety, cytokine biomarkers

## Abstract

**Purpose:**

A phase 3 randomized study (COG ANBL0032) demonstrated significantly improved outcome by adding immunotherapy with ch14.18 antibody to isotretinoin as post-consolidation therapy for high-risk neuroblastoma (NB). This study, ANBL0931, was designed to collect FDA-required safety/toxicity data to support FDA registration of ch14.18.

**Experimental design:**

Newly diagnosed high-risk NB patients who achieved at least a partial response to induction therapy and received myeloablative consolidation with stem cell rescue were enrolled to receive six courses of isotretinoin with five concomitant cycles of ch14.18 combined with GM-CSF or IL2. Ch14.18 infusion time was 10–20 h per dose. Blood was collected for cytokine analysis and its association with toxicities and outcome.

**Results:**

Of 105 patients enrolled, five patients developed protocol-defined unacceptable toxicities. The most common grade ≥ 3 non-hematologic toxicities of immunotherapy for cycles 1–5, respectively, were neuropathic pain (41, 28, 22, 31, 24%), hypotension (10, 17, 4, 14, 8%), allergic reactions (ARs) (3, 10, 5, 7, 2%), capillary leak syndrome (1, 4, 0, 2, 0%), and fever (21, 59, 6, 32, 5%). The 3-year event-free survival and overall survival were 67.6 ± 4.8% and 79.1 ± 4.2%, respectively. AR during course 1 was associated with elevated serum levels of IL-1Ra and IFNγ, while severe hypotension during this course was associated with low IL5 and nitrate. Higher pretreatment CXCL9 level was associated with poorer event-free survival (EFS).

**Conclusion:**

This study has confirmed the significant, but manageable treatment-related toxicities of this immunotherapy and identified possible cytokine biomarkers associated with select toxicities and outcome. EFS and OS appear similar to that previously reported on ANBL0032.

## Introduction

Immunotherapy using anti-glycolipid disialoganglioside (GD2) antibody combined with cytokines has become the standard treatment in North America for patients with high-risk neuroblastoma (NB) who have achieved at least a partial remission following intensive induction and consolidation (1). Induction treatment consists of 5–6 cycles of multiagent chemotherapy combined with surgery followed by the consolidation phase that includes single or tandem autologus stem cell transplant with subsequent radiotherapy. Several anti-GD2 antibodies have been tested for clinical use including murine 3F8, chimeric 14.18 (ch14.18, dinutuximab), humanized 14.18 (hu14.18K322A), and humanized 14.18 fused to interleukin-2 (hu14.18-IL2) (2–5). Ch14.18 is an anti-GD2 monoclonal antibody, which is a chimeric construct composed of the variable region heavy and light chain genes of the murine mAb14.G2a and the human constant region genes for heavy chain IgG1 and light chain kappa (6). The primary objective of this study was to comprehensively define the safety profile of ch14.18 when administered with cytokines and isotretinoin in high-risk NB patients after completing standard induction chemotherapy and autologous stem cell transplant (ASCT) and generate data required to support the Biological License Application (BLA) for ch14.18 with the Food and Drug Administration (FDA).

Cytokine release often occurs during antibody-based immunotherapeutics and is commonly associated with infusion reactions and other toxicities. Therapeutic IL-2 not only induces immune cell stimulation but may also induce hypotension and capillary leak syndrome. This is believed to be mediated by nitric oxide (NO) directly or indirectly *via* induction of tumor necrosis factor alpha (TNFα) and interferon gamma (IFNγ) or other proinflammatory cytokines such as IL-6 (7–9). Cytokine release in response to other immunotherapies is common and believed to be responsible for associated toxicities (10). Cytokines have also been implicated in patient survival, with increased IL-6 levels at diagnosis associated with poor outcome in numerous cancers including NB (10, 11). However, the relationship of cytokine levels with outcome from immunotherapy has never been investigated. Thus, serum cytokine profiles during ch14.18 immunotherapy may be able to predict toxicities and/or outcome of the immunotherapy and were thus investigated as part of this study.

## Materials and Methods

### Patient Population

All NB patients categorized as high-risk at the time of diagnosis, and met the International Neuroblastoma Response Criteria (INRC) for complete response, very good partial response (PR), or PR for primary site, soft tissue, bone metastases at their pre-ASCT evaluation at study entry were eligible [(12), described in online Appendix]. High-risk patients were International Neuroblastoma Staging System (INSS) stage 4 greater than 18 months of age, INSS stage 4 with MYCN amplification, regardless of age, INSS stage 4 between ages of 12 and 18 months with unfavorable histology and/or diploid tumor DNA content, INSS stage 3 with amplified MYCN, regardless of age, INSS stage 3 and unfavorable histology greater than 18 months of age, INSS stage 2 with MYCN amplification regardless of age. In addition, all patients must have completed therapy including intensive induction chemotherapy followed by myeloablative consolidation with ASCT and radiotherapy, including enrollment onto contemporary clinical trials within the Children’s Oncology Group or New Advances in Neuroblastoma Research (regimen specifics included in Appendix, online only). No more than 9 months from the date of starting the first induction chemotherapy to the date of ASCT was allowed. Patients had to be enrolled no later than Day 100 after ASCT infusion (or day 100 from second stem cell infusion if tandem transplant). Patients had to be enrolled after completion of radiotherapy post-ASCT, and after completion of tumor assessment post-radiotherapy. There was no age restriction. Patients who had received prior anti-GD2 therapy were excluded. Other organ-specific and inclusion/exclusion criteria are provided in the Appendix (online only).

Written informed consent was obtained from parents or legal guardians. Patients were treated at thirty Children’s Oncology Group institutions on a protocol approved by the institution’s local Institutional Review Board (IRB) or National Cancer Institute (NCI)-sponsored pediatric central institutional review board (NCT01041638; see Appendix for the list of institutions, online only).

### Study Design

**Figure 1:**
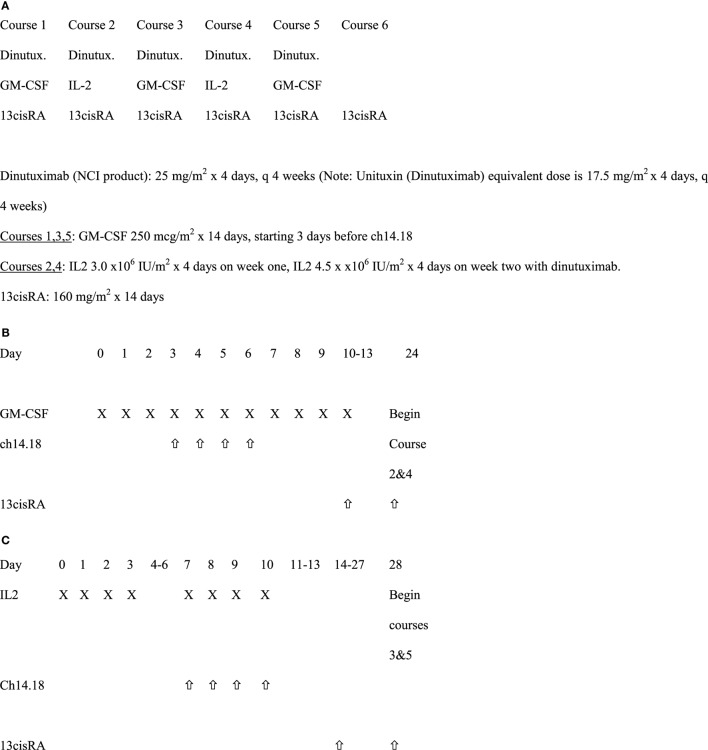
Immunotherapy treatment schema. **(A)** Schedule of overall dinutuximab, GM-CSF, IL2, and 13cisRA. **(B)** Treatment schema for courses 1, 3, and 5 with GM-CSF (28 days per course). **(C)** Treatment schema for courses 2 and 4 with IL2.

All patients received six courses of isotretinoin (ISOT). For the first five of these courses, patients also received ch14.18 plus cytokines, with ch14.18 and sargramostim (granulocyte macrophage-colony stimulating factor, GM-CSF) administered in Courses 1, 3, and 5, and ch14.18 with aldesleukin (IL-2) given in Courses 2 and 4 (Figure 1) Ch 14.18 was administered every 28 days, as described previously (1).

Toxicities were graded according to the Common Terminology Criteria for Adverse Events (version 4.0). Grades 1 through 5 toxicities were captured. Unacceptable toxicities were defined as: Grade ≥ 4 allergic reaction (AR), anaphylaxis, Grade ≥ 4 capillary leak syndrome, Grade ≥ 3 peripheral motor neuropathy with duration ≥ 2 weeks, and Grade ≥ 4 pain, requiring narcotics/lidocaine and persisting ≥ 4 days after the end of ch14.18 infusion. AR was defined to include bronchospasm, stridor, wheezing, urticarial, and anaphylaxis. Special attention was placed on toxicities resulting from direct neuronal toxicity or from immune/cytokine reactions extrapolated from similar data based on ANBL0032 (NCT01418495). Required pretreatment and on-study observations were used to monitor toxicity and criteria for dose modifications (Appendix, online only). Tumor response was reported using INRC.

### Drug Supply and Administration

Ch14.18 was supplied by the National Cancer Institute and administered to hospitalized patients intravenously over a minimum of 10 h at 25 mg/m^2^/day for four consecutive days every 28 days. This dose is the equivalent of the currently FDA approved dinutuximab dose of 17.5 mg/m^2^/day. Ch14.18 infusion was started at 1.25 mg/m^2^/h for the first 0.5 h, then, increased to 2.5 mg/m^2^/h for the remainder of the dose, if tolerated, to be administered over ≥10 h. The maximum infusion time from initiation of ch14.18 was 20 h even if the total dose had not been administered in that timeframe. Patients were pre-medicated with diphenhydramine or hydroxyzine, acetaminophen and recommended to be on intravenous (IV) narcotics as a loading dose immediately prior to ch14.18 administration followed by continuous IV infusion narcotics. During courses 1, 3, and 5, commercially available GM-CSF was administered either subcutaneously (strongly recommended) or IV over 2 h at 250 µg/m^2^/day for 14 days. Ch14.18 was started on the fourth day of GM-CSF injections, 1 h after GM-CSF and immediately following a 10 ml/kg IV bolus of normal saline each day. During course 2 and 4, commercially available aldesleukin (IL-2) at 3 Million International Units/m^2^/day was administered by IV continuous infusion for 96 h. Seven days later, patients received an additional 4.5 Million IU/m^2^/day of aldesleukin administered by IV continuous infusion over 96 h along with simultaneous administration of ch14.18. Isotretinoin at 80 mg/m^2^/dose (2.67 mg/kg/dose if weight ≤ 12 kg) was administered by mouth twice daily (BID) for 14 days, every 28 days starting on day 11 for course 1, day 10 for courses 3 and 5, and day 14 for courses 2 and 4. Patients received only isotretinoin for course 6 (same dose as in previous courses) starting 14 days after completion of course 5.

The use of corticosteroids and other immunosuppressive medications were prohibited, except for life-threatening conditions (i.e., life-threatening AR, including anaphylaxis, bronchospasm, and stridor) unresponsive to other measures. Other supportive care measures are listed in the Appendix (online only).

### Pharmacokinetic Studies

Plasma ch14.18 levels were quantified using an electrochemiluminescence immunoassay (BioAgilytix, Durham, NC, USA) as previously described (13). Samples were obtained before starting GM-CSF and after ch14.18 in Courses 1 and 5 and prior to IL-2 and after ch14.18 and IL-2 in Course 4. A final sample was obtained within 2 weeks after the last dose of isotretinoin in Course 6.

### Cytokine and Nitrate Analysis

Heparinized blood and serum samples were collected before starting immunotherapy and just before the fourth doses of ch14.18 infusion on courses 1 (days −1 and 6) and 4 (days 80 and 90) and shipped by overnight courier for lab analyses. Most analytes were measured using SimplePlex assays (ProteinSimple, San Jose, CA, USA) except the following: IL-6 by the Meso Scale Discovery (MSD, Rockville, MD, USA) IL-6 assay, IL-1 receptor antagonist (IL-1Ra) by the R&D Systems assay (R&D Systems, Minneapolis, MN, USA), and nitrate by the R&D Systems Parameter Total Nitric Oxide and Nitrate/Nitrite Kit.

### Human Anti-Chimeric Antibody (HACA) Studies

A validated MSD electrochemiluminescent assay was used to measure HACA (BioAgilytix, Durham, NC, USA). Methodological details are included in Appendix (Online only).

### Statistical Analysis

In accordance with the protocol, analyses were performed as intent-to-treat. Toxicity data were summarized using proportions with two-sided 95% Wilson (score) confidence intervals. For selected toxicities of interest, the median, minimum, and maximum duration were calculated. Protocol monitoring rules for unacceptable toxicity (two-stage design) and toxic death were applied. McNemar’s test for paired data was used to compare the incidence of toxicities between GM-CSF (cycles 1, 3, and 5) and IL-2 (cycles 2 and 4) containing courses in patients with at least one course of each. Event-free survival (EFS) and overall survival (OS) curves were generated using the methods of Kaplan and Meier, with standard errors per Peto et al. (14, 15). For EFS, the time to event was calculated from study enrollment until the first occurrence of relapse, progressive disease, secondary malignancy, death, or until last contact if no event occurred. For OS, it was time from enrollment until death, or until last contact with the patient. Cytokine levels during courses were compared by Wilcoxon signed-rank test comparison of paired samples: day −1 versus day 6 and day 80 versus day 90. A *p*-value of <0.0036 was considered statistically significant at level 0.05 after Bonferroni correction for the 14 cytokine compared at each course. A two-sided Mann–Whitney *U*-test was used to compare analyte levels in patients experiencing a vascular toxicity (hypotension or capillary leak) or AR (anaphylaxis, urticaria, wheezing, stridor, bronchospasm, and generally diagnosed AR) versus no AR and was considered statistically significant at *p* ≤ 0.05.

## Results

### Patient Characteristics

One hundred and five patients were enrolled. One patient withdrew consent prior to the initiation of therapy, but remains included in these intent-to-treat analyses. Ninety-three percent of the patients were less than 12 years old. Patient characteristics are summarized in Table 1

**Table 1:** Characteristics of patients enrolled on COG ANBL0931 (*n* = 105).

Age, years	
Median	4.1
Range	1.1–27.5
Sex	
Male	59 (56.2%)
Female	46 (43.8%)
Race	
White	82 (78.1%)
Black	10 (9.5%)
Other	13 (12.4%)
Ethnicity	
Hispanic	9 (8.6%)
Other	96 (91.4%)
Histology	
Favorable	4 (5.5%)
Unfavorable	69 (94.5%)
Unknown	32
INSS stage	
Stage 1^a^	2 (2.5%)
Stage 2^b^	1 (1.2%)
Stage 3	15 (18.5%)
Stage 4	63 (77.8%)
Unknown	24
*MYCN* status	
Non-amplified	40 (57.1%)
Amplified	30 (42.9%)
Unknown	35
Number of ASCTs prior to study	
One	88 (83.8%)
Two	17 (16.2%)
Disease status at study entry	
Complete response	29 (27.6%)
Very good partial response (PR)	36 (34.3%)
PR	40 (38.1%)

*^a^MYCN amplified tumor (*n* = 1), progression to metastatic disease meeting high-risk criteria during follow-up (*n* = 1)*.

*^b^MYCN amplified tumor*.

### Toxicities

Five patients developed protocol-defined unacceptable toxicities and came off study: four Grade 4 AR and one sudden death (Table 2). The pre-specified monitoring rule for too many unacceptable toxicities was not met. Sudden death occurred in one patient who had been clinically stable while awaiting start of course 2 of immunotherapy. The patient experienced sudden onset of abdominal pain and subsequent cardiac arrest and unable to be resuscitated by paramedics. No autopsy was performed. Three more patients discontinued the study as per parents/physician choice secondary to immunotherapy-related toxicities.

**Table 2:** Toxicities defined as “Unacceptable” according to COG protocol ANBL0931 (*n* = 105 patients enrolled).

Course	Number of patients with unacceptable toxicities	Description of the unacceptable toxicity
Course 1 (*n* = 104)	2	Grade 4 allergic reaction (AR)/anaphylaxis (*n* = 1); grade 5 cardiac arrest (*n* = 1)
Course 2 (*n* = 100)	1	Grade 4 AR
Course 3 (*n* = 98)	1	Grade 4 AR
Course 4 (*n* = 90)	1	Grade 4 anaphylaxis
Course 5 (*n* = 88)	0	None
Course 6 (*n* = 81)	0	None

The proportion of patients experiencing clinically relevant toxicities, all grades, across all cycles of therapy are summarized in Table 3 (Grade 1 and 2 toxicities provided in Table S1 in Supplementary Material). The most common Grade 3 or higher non-hematologic toxicities of immunotherapy were neuropathic pain and fever, followed by, hypotension, AR, and capillary leak syndrome (Table 4). Of the 104 patients who received course 1, 103 (99%) experienced at least one Grade 3 or greater toxicity (data not shown). Pain occurred more frequently during Course 1, then, at lower rates thereafter (Tables 3 and 4). There was not a statistically significant difference for AR, capillary leak syndrome and hypotension between GM-CSF vs IL-2 courses. Although not statistically significant, capillary leak syndrome and grades 3–4 hypotension occurred at higher rates during the IL-2 courses. The median duration of select adverse events is presented in Table 5. Most toxicities resolved within 3 days with the exception of the rare ocular and hypertension toxicity (Table 5). Dose reduction of ch14.18 was reported in 45 patients (43.27%), i.e., these patients received less than 90% of the intended dose at some point during courses 1–5. Of the 45 patients with dose reductions, ARs, capillary leak, and hypotension were the main indications. Of the 104 patients who received treatment 79 patients received ≥ 90%, 8 patients ≥ 80 to <90%, 9 patients ≥70 to <80%, 1 patient ≥ 60 to <70%, 3 patients ≥ 50 to <60%, and 4 patients received <50% of the cumulative intended dose of ch14.18. The use of morphine, fentanyl, meperidine, hydromorphone, and ketamine were reported in 100 patients at some point during the study. In 20 of the 104 patients (19.2%), GM-CSF was administered intravenously instead of subcutaneously.

**Table 3:** Proportion of patients with Grades 1–5 toxicities attributed to ch14.18 on COG study ANBL0931.

	Pain (%)	Allergic reactions (%)	Capillary leak syndrome (%)	Hypotension (%)	Fever (%)	Ocular toxicity (%)
Course 1 (*n* = 104)	93	25	24	60	91	2
Course 2 (*n* = 100)	78	28	39	64	92	3
Course 3 (*n* = 98)	79	16	14	58	69	3
Course 4 (*n* = 90)	77	21	33	69	87	1
Course 5 (*n* = 88)	70	13	17	52	67	1
Course 6 (*n* = 81)	26	0	0	14	19	0

**Table 4:** Proportion of patients (95% confidence interval) with clinically significant Grade 3 and 4 toxicities attributed to ch14.18 on COG study ANBL0931 (courses 1, 3, and 5 contained GM-CSF and Courses 2 and 4 contained IL-2).

	Pain	Allergic reactions	Capillary leak syndrome	Hypotension	Fever
Course 1 (*n* = 104)	41% (32.4, 51.0%)	3% (0.9, 8.1%)	1% (0.2, 5.2%)	10% (5.3, 16.8%)	21% (14.4, 30.0%)
Course 2 (*n* = 100)	28% (20.1, 37.5%)	10% (5.5, 17.4%)	4% (1.6, 9.8%)	17% (10.9, 25.6%)	59% (49.2, 68.1%)
Course 3 (*n* = 98)	22% (15.3, 31.7%)	5% (2.2, 11.4%)	0% (0.0, 3.8%)	4% (1.6, 10.0%)	6% (2.8, 12.7%)
Course 4 (*n* = 90)	31% (22.5, 41.3%)	7% (3.1, 13.8%)	2% (0.6, 7.7%)	14% (8.6, 23.2%)	32% (23.5, 42.4%)
Course 5 (*n* = 88)	24% (16.2, 33.7%)	2% (0.6, 7.9%)	0% (0.0, 4.2%)	8% (3.9, 15.5%)	5% (1.8, 11.1%)

**Table 5:** Median duration (in days) of select adverse events within the subset of patients who experienced those events^a^.

	Courses 1, 3, or 5 (with GM-CSF) (*n* = 104)		Courses 2 or 4 (with IL-2) (*n* = 100)		Anytime on treatment	
Toxicities	*N* with toxicity	Median duration (days)	*N* with toxicity	Median duration (days)	*N* with toxicity	Median duration (days)
Neuropathic pain	54	2.0	38	2.0	64	2.5
Ocular toxicities^cb^	14	3.5	6	69.0	19	5.0
Anatomical pain^d^	54	2.0	39	2.0	64	3.0
Capillary leak syndrome	1	2.0	6	2.5	7	2.0
Allergic reaction	8	1.5	13	2.0	17	2.0
Hypertension	1	238.0	2	7.0	3	13.0
Fever	28	1.0	63	2.0	70	2.0

*^a^For a given adverse event, the longest duration of an event was selected per patient per treatment course. Only Grades 3–4 were used in calculations unless otherwise specified (the Grade 5 cardiac arrest has been excluded from this table)*.

*^b^One ocular toxicity was ongoing and had no date of resolution. This record is omitted from calculations*.

*^c^All grades were used for calculations*.

*^d^See Table S1 in Supplementary Material for the description of anatomical pain*.

### Pharmacokinetics

The mean ch14.18 concentration appeared to be similar during the peak of the three courses studied (first, third, and fourth), with mean concentrations of 5,965, 4,650, and 5,907 ng/ml, respectively (Table 6). Ch14.18 was still detectable at its trough time points, just prior to starting the third and fourth courses, with mean concentrations of 844 and 730 ng/ml, respectively.

**Table 6:** Summary of dilution corrected mean of ch14.18 concentration values (ng/mL).

Study day	*N* (patients)	Mean	SD	Median	Min, max
6 (peak)	80	5,965	2,463	5.761	1,233, 13,380
80 (trough)	78	844	1,598	341	6, 10,910
90 (peak)	71	4,650	2,263	4,506	34, 9,611
111 (trough)	66	730	1,468	362	8, 11,630
118 (peak)	63	5,907	2,550	5,844	77, 15,630
Completion of study (trough)	66	178	188	126	1, 890

### HACA Response

A total of 533 samples were analyzed for HACA from 103 patients. Eight patients had a confirmed positive HACA response. All HACA positivity was detected during and after course 3. No dose modification was done based on HACA development.

### Cytokine Profiles and Association With ARs

**Figure 2:**
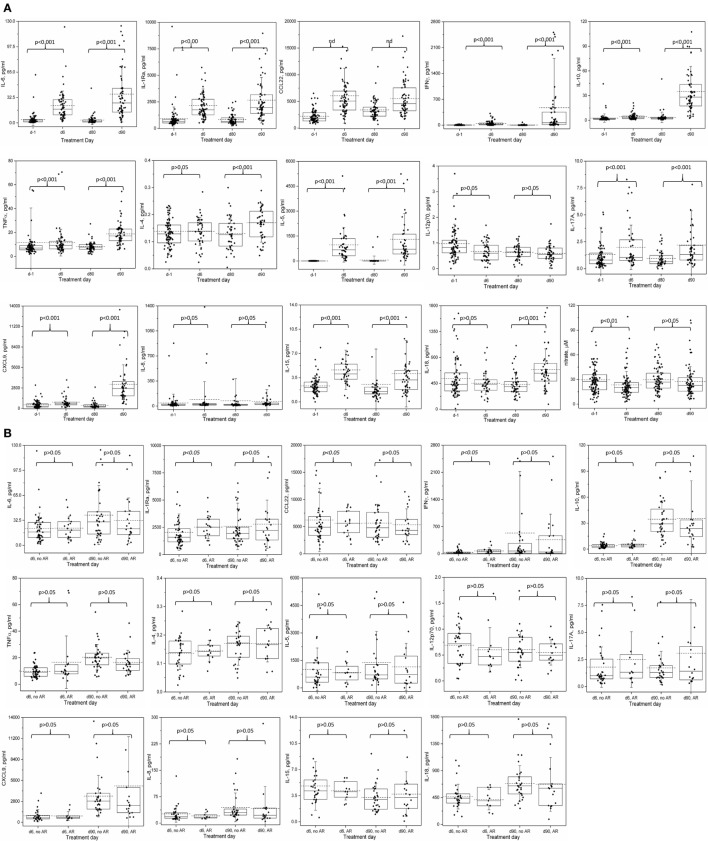
Cytokine and nitrate levels during course 1 and 4 of immunotherapy: **(A)** changes in serum cytokine and nitrate levels during Immunotherapy. **(B)** Relationship between allergic reaction of any grade and serum cytokine levels on immunotherapy. Serum samples were collected before starting immunotherapy and just before the fourth doses of ch14.18 infusions on courses 1 (days 1 and 6) and 4 (days 80 and 90), and analyzed as described for cytokine and nitrate levels. Box represents 25–75% distribution, error bars represent SD, solid line is the median, dashed line is the mean. Boxes and values shown include any not-shown outliers unless otherwise mentioned. **(A)** Comparisons are by Wilcoxon signed-rank test of paired samples: day −1 versus day 6 and day 80 versus day 90. A *p*-value of < 0.0036 is considered statistically significant at level 0.05 and are Bonferroni corrected for the 14 cytokine comparisons at each course, or *p* < 0.05 for nitrate. Values of outliers excluded from the graph are: d-1: IL-6 (447.4 and 416.7 pg/ml), tumor necrosis factor alpha (268.5 pg/ml), IL-4 (0.78 pg/ml), IL-12p70 (5.48 pg/ml), IL-17A (22.4 pg/ml), IL-8 (31,818 pg/ml and also excluded from the mean, median and statistical determinations); d6: IL-6 (618 pg/ml), IL1-Ra (11,489.1 pg/ml), CCL22 (39,956.7 pg/ml), IL-15 (19.2 pg/ml); d80: IL-6 (862.0 pg/ml), IL-8 (2,185.5 pg/ml), IL-15 (33.7 pg/ml and 17.1 pg/ml); d90: IL-6 (187.1 pg/ml), IL1-Ra (13,329.9 pg/ml), IFNγ (9,591.9 pg/ml), IL-10 (193.7 pg/ml), IL-4 (0.47 pg/ml), IL-5 (7,500.9 pg/ml), IL-17A (20.7 pg/ml), CXCL9 (24,172.7 and 19,116.1 pg/ml), IL-15 (33.4 pg/ml). **(B)** Two-sided Mann–Whitney *U*-test comparison of no AR versus any AR; values are not adjusted for multiple comparisons. Values of outliers excluded from the graph are: d6, no AR: IL1-Ra (11,489.1 pg/ml), CCL22 (39,956.7 pg/ml), IL-15 (19.2 pg/ml); d6 AR: IL-6 (618 pg/ml); d90, no AR: IL1-Ra (13,329.9 pg/ml), IFNγ (9,591.9 pg/ml), IL-4 (0.47 pg/ml), IL-5 (7,500.9 pg/ml), IL-15 (33.4 pg/ml); d90 AR: IL-10 (193.7 pg/ml), IL-17A (20.7 pg/ml), CXCL9 (24,172.7 and 19,116.1 pg/ml). Notes: IL-1Ra: interleukin-1 receptor antagonist; many CCL22 values were out of the range of the multiplex assay and were extrapolated. Thus, the CCL22 data and analyses should be considered qualitative.

After correcting for multiple comparisons, the average levels of 9 of 13 cytokines on course 1, and 11 of 13 on course 4 were significantly elevated with respect to the pre-treatment values (Figure 2A; Table S2 in Supplementary Material). IL-5 exhibited the most dramatic rise during immunotherapy. On the other hand, IL-4 and IL-18 on course 1 and IL12p70 and IL-8 on both courses did not change significantly during treatment.

The average cytokine levels of patients with AR were indistinguishable from the overall population, with no significant differences in the levels of the cytokines between the “non-AR” versus the “any AR” population of patients, except for course 1 (day 6) IL-1Ra (2,043.6 vs 2,522.3 pg/ml, *p* = 0.02) and IFNγ (35.9 vs 73.8 pg/ml, *p* = 0.02), though neither was significant at course 4 (day 90) (Figure 2B; Table S2 in Supplementary Material). Similarly, when levels for each of the cytokines were compared for patients with no or Grade 1 AR versus patients with Grade ≥ 2 AR, no associations were identified (data not shown). At each time point, we then investigated the 14 individual patients with the highest level of each of the 14 cytokines. At day 6, the patients with “extreme” cytokine levels were as likely to be associated with the non-AR group as with the AR group (7 of 14 cytokines in each group). At day 90, only 3 of the patients with the extreme cytokine value had an AR; the patients with the highest levels of IL-10 and IL-17A experienced grade 1 toxicities (urticaria and AR, respectively), while the patient with the highest levels of CXCL9 experienced grade 3 urticaria. It should be pointed out that any toxicity that occurred between the start of a given course and before the fourth dose of ch14.18 (day 6 for course 1 or day 90 for course 4), when blood was drawn, were considered for correlations with cytokine levels.

Among the patients with anaphylaxis, a blood sample was available from only one patient (during Course 4, Figure 2B; Table S2 in Supplementary Material) taken the day prior to the documentation of anaphylaxis. Most of the cytokines levels on day 90 in this patient were ≤2-fold different from the average levels of all course 4 AR patients. One exception was IL-17A, a cytokine known to be associated with food allergy (16). The IL-17A levels in this patient (4.02 ± 0.14) were higher than 29 of 32 (91%) patients who did not have any AR. Furthermore, of patients who had similar (±20%, *N* = 4) or higher (>20%, *N* = 3) IL-17A levels, four of these patients also experienced AR, though the patient harboring the highest IL-17A levels of all patients (20.73 pg/ml) experienced only a grade 1 AR. In contrast, IFNγ in this anaphylaxis patient (32.9 ± 0.3 pg/ml) was 11–16 fold *lower* than the average of all d90 patients, AR average and non-AR group average (474.7 ± 169.1, 365.9 ± 139.8, and 534.6 ± 251.5, respectively, Figure 1B; Table S2 in Supplementary Material), though 9 other AR patients and 14 non-AR patients had even lower IFNγ levels.

### Cytokine and Nitrate Profiles and Association With Vascular Disorders

As IL-5 levels were dramatically elevated on both courses investigated, we explored a possible relationship of this cytokine and the other analytes implicated with vascular disorders for associations with clinically significant (≥grade 3) hypotension (Table 7), or any grade capillary leak (Table 8). During course 1, hypotension was associated with *lower* IL-5 levels (*p* = 0.018), though, none of the associations met the required level for statistical significance when adjusted for the four analytes compared (*p* < 0.0125). Nitric oxide, as measured by the more stable precursor nitrate, showed a small but significant decrease in patients with ≥ grade 3 hypotension during course 1, contrary to the expected association of *higher* nitrate levels with hypotension (Table 7). No association of any of the cytokines or nitrate with hypotension was observed during the IL-2 containing course 4.

**Table 7:** Relationship of cytokine and nitrate levels with severe hypotension in ANBL0931 patients.

	Course 1, day 6			Course 4, day 90		
Cytokine or nitrate	None or ≤ gr2 hypotension	hypotension ≥ gr3	p-Value	None or ≤ gr2 hypotension	hypotension ≥ gr3	p-Value
IL-5 (pg/ml)	*N* = 42	*N* = 4	*p* = 0.018	*N* = 39	*N* = 7	*p* > 0.05
Mean ± SE (range)	1,043.1 ± 162.7 (27.5–5,114.5)	148.0 ± 51.9 (12.3–264.9)		1,224.7 ± 211.1 (28.5–5,238.4)	1,752.0 ± 982.2 (3.6–7,500.9)	

Tumor necrosis factor alpha (pg/ml)	*N* = 61	*N* = 7	*p* > 0.05	*N* = 54	*N* = 8	*p* > 0.05
Mean ± SE (range)	11.8 ± 1.5 (2.5–70.6)	8.6 ± 1.4 (4.7–13.6)		19.4 ± 1.3 (5.7–54.4)	15.3 ± 2.1 (7.0–26.0)	

IFNγ (pg/ml)	*N* = 61	*N* = 7	*p* > 0.05	*N* = 54	*N* = 8	*p* > 0.05
Mean ± SE (range)	48.2 ± 8.2 (1.2–317.2)	26.2 ± 9.9 (2.2–61.0)		354.4 ± 89.7 (0.6–2,511.0)	1,286.5 ± 1,187.9 (3.1–9,591.9)	

IL-6 (pg/ml)	*N* = 63	*N* = 7	*p* > 0.05	*N* = 55	*N* = 8	*p* > 0.05
Mean ± SE (range)	31.8 ± 9.8 (1.7–618. 6)	19.1 ± 4.7 (2.5–35.5)		38.3 ± 5.1 (0.9–187.1)	26.2 ± 5.3 (1.0–54.7)	

Nitrate $\left(NO_{3}^{-}\right)$ (μM)	*N* = 81	*N* = 8	*p* = 0.04	*N* = 67	*N* = 10	*p* > 0.05
Mean ± SE (range)	24.2 ± 1.9 (3.5–27.8)	13.9 ± 3.0 (2.8–27.8)		28.3 ± 2.4 (5.9–102.0)	21.0 ± 2.7 (7.6–34.3)	

*Cytokines or nitrate during course 1 and course 4 of immunotherapy for association with clinically significant (≥grade 3) hypotension. Mann–Whitney *U*-test comparisons of cytokines were considered significant at a *p*-value of <0.0125 after Bonferroni correction for the four cytokines measured at each course. Nitrate levels were considered significant at *p* = 0.05*.

**Table 8:** Relationship of cytokine and nitrate levels with capillary leak in ANBL0931 patients.

	Course 1, day 6			Course 4, day 90		
Cytokine or nitrate	No capillary leak^a^	Capillary leak ≥ gr2^b^	*p*-value^c^	No capillary leak^a^	Capillary leak ≥ gr2^d^	*p*-value^c^
IL-5 (pg/ml)	*N* = 30	*N* = 16	*p* > 0.05	*N* = 25	*N* = 21	*p* > 0.05
Mean ± SE (range)	903.3 ± 133.4 (27.5–2,787.7)	1,081.4 ± 369.4 (12.3–5,114.5)		757.9 ± 105.8 (3.6–2,175.8)	1,956.1 ± 450.2 (28.5–7,500.9)	

IL-6 (pg/ml)	*N* = 48	*N* = 22	*p* > 0.05	*N* = 39	*N* = 24	*p* > 0.05
Mean ± SE (range)	21.0 ± 2.0 (1.7–72.6)	51.4 ± 27.6 (2.5–618.6)		34.8 ± 5.0 (1.0–124.2)	40.0 ± 8.7 (0.9–187.1)	

Tumor necrosis factor alpha (TNFα) (pg/ml)	*N* = 46	*N* = 22	*p* > 0.05	*N* = 38	*N* = 24	*p* > 0.05
Mean ± SE (range)	9.6 ± 0.7 (2.5–23.7)	15.4 ± 0.3 (3.0–70.6)		17.6 ± 1.0 (6.0–35.7)	20.8 ± 2.6 (5.7–54.4)	

IFNγ (pg/ml)	*N* = 46	*N* = 22	*p* > 0.05	*N* = 60	*N* = 24	*p* > 0.05
Mean ± SE (range)	47.7 ± 10.0 (1.2–317.2)	42.2 ± 10.2 (1.4–194.8)		628.6 ± 266.5 (3.1–9,591.9)	231.0 ± 103.8 (0.6–2,459.6)	

Nitrate $\left({\text{NO}}_{3}^{-}\right)$ (μM)	*N* = 65	*N* = 24	*p* > 0.05	*N* = 48	*N* = 29	*p* > 0.05
Mean ± SE (range)	23.2 ± 2 (2.8–106.41)	23.6 ± 3.3 (3.8–79.9)		25.8 ± 2.5 (5.9–102.0)	29.8 ± 3.8 (7.2–98.7)	

*^a^There were no grade 1 capillary leak diagnoses*.

*^b^One patient was diagnosed with grade 3 while the rest were grade 2. Compared to the group as a whole (see Table S2 in Supplementary Material), this patients levels of IL-5 were the lowest, IL-6 the second lowest and TNFα ninth lowest. IFNγ and nitrate appeared similar to the group as a whole (#26 for IFNγ and #69 for nitrate)*.

*^c^Test of association of cytokine or nitrate levels with capillary leak (none versus grade ≥ 2) using a two-sided Mann–Whitney *U*-test at a *p*-value of < 0.05 without correction for multiple comparisons*.

*^d^Two patients were diagnosed with grade 3 capillary leak at this time point while the rest were grade 2*.

Capillary leak incidence varied between 0 and 4% during different courses (Table 4). Nevertheless, in an exploratory fashion, we compared the cytokine and serum nitrate profile among patients experiencing capillary leak of grade ≥ 2 and those without capillary leak (Table 8). At course 1, IL-6 was the only cytokine of the four (TNFα, IFNγ, IL-6, IL-5) with higher levels in the capillary leak population (51.4 ± 27.6, *N* = 22), than the population without capillary leak (21.0 ± 2.0, *N* = 48, *p* > 0.05) though not statistically significant. Similarly, no statistically significant difference was identified for nitrate levels between the none vs grade ≥ 2 capillary leak subgroups. During course 4, average IL-5 levels were increased and IFNγ levels were decreased in the none vs grade ≥ 2 capillary leak subgroups, though again the groups were not statistically different (Table 8). In contrast to hypothesized high levels of cytokines, the patient who developed course 1 grade 3 capillary leak harbored day 6 levels of IL-5 that were the lowest of all patients, IL-6 the second lowest and TNFα ninth lowest of the entire analyzed population. On the other hand, serum IFNγ and nitrate levels appeared similar to the overall patient cohort (26th lowest of 68 patient samples for IFNγ and 69th lowest of 89 patient samples for nitrate). For the two patients with grade 3 capillary leak during course 4, we observed a cytokine expression pattern similar to that observed in the patient who developed capillary leak during course 1, with very low IL-5 (second and fifth lowest), IL-6 (fourth and eleventh lowest), and TNFα (sixth and thirteenth lowest). However, in contrast to day 6, IFNγ levels in these two patients were the lowest and second lowest of all patients measured. Among the 56 unique patients who reported Grade 2 or 3 capillary leak syndrome, 32 patients reported in more than one course. Fourteen patients reported in two, 4 reported in three, 9 reported in four, and 5 reported in five courses.

### Disease Outcome

**Figure 3:**
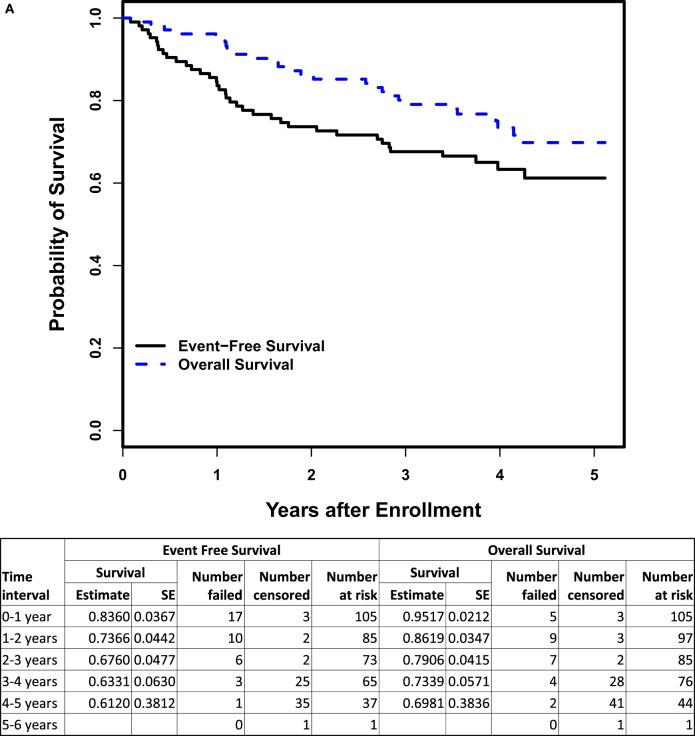

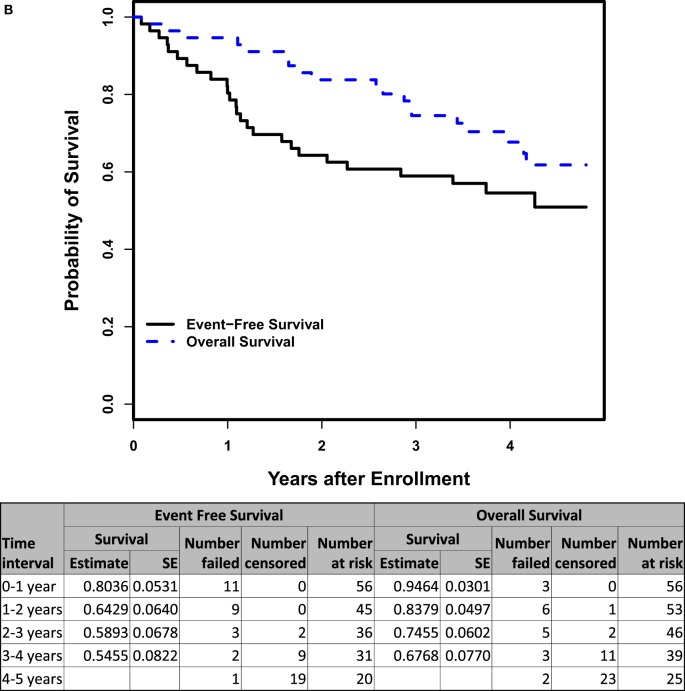
**(A)** Event-free and overall survival (OS) from time of enrollment for patients on COG ANBL0931 (*n* = 105). **(B)** Event-free and OS from time of enrollment for patients > 18 months of age with INSS Stage 4 disease on COG ANBL0931 (*n* = 56).

The 3-year EFS and OS rates (*n* = 105) were 67.6 ± 4.8 and 79.1 ± 4.2%, respectively (Figure 3A). For the subset of patients > 18 months of age with INSS Stage 4 disease (*n* = 56), the 3-year EFS and OS rates were 58.9 ± 6.8 and 74.6 ± 6.0%, respectively (Figure 3B). There was no difference in EFS between patients who received ≥ 90% (*n* = 79; 3-year EFS: 70.3 ± 5.4%) vs. <90% (*n* = 25; 3-year EFS: 57.6 ± 10.4%) of the cumulative ch14.18 dose intended (log-rank *p*-value = 0.1486). There was no significant difference in the progressive disease rate between the lowest and highest quartiles when analyzed based on the cumulative ch14.18 received per patient (chi-square *p*-value = 0.7306).

### Cytokines and Outcome

**Figure 4:**
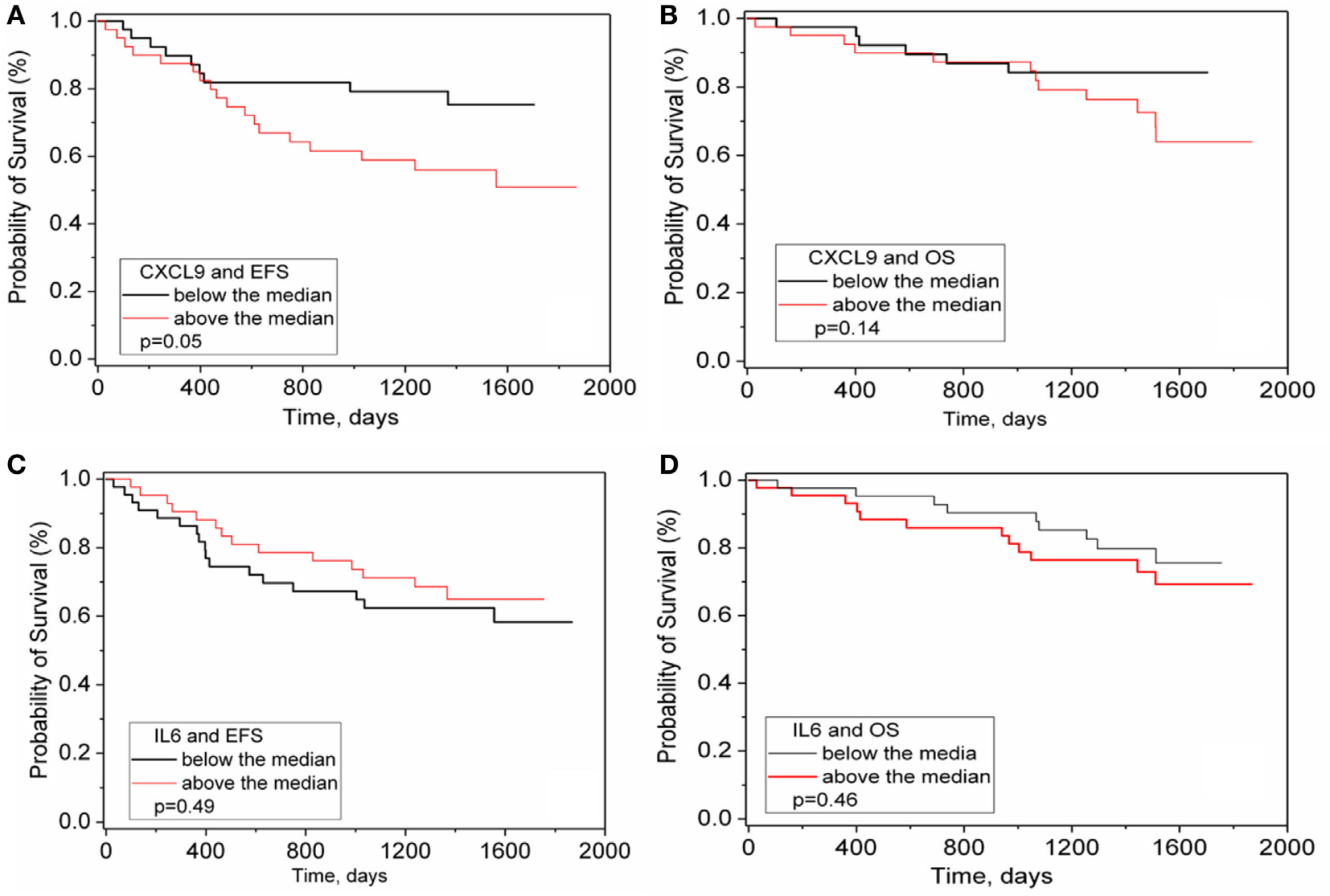
CXCL9 and IL-6 versus survival in immunotherapy treated patients. CXCL9 **(A,B)** and IL-6 **(C,D)** were measured in the serum of ANBL0931 patient samples obtained at pretreatment to course 1. Patient values were sorted as to above or below median levels of all patients and a Kaplan Meier survival analysis for event-free survival **(A,C)** and OS **(B,D)** assessed and significance assessed by Log Rank test.

Levels of IL-6 and other cytokines prior to initial chemotherapy have been associated with outcome in numerous cancers including NB (10, 11). However, among the 15 analytes measured at four treatment time points in two courses of maintenance phase immunotherapy, only CXCL9 at day −1 showed an association with EFS (*p* = 0.05, not Bonferroni corrected, Figures 4A–B). No association with OS was observed nor was there any outcome association with *changes* in levels of any of the analytes including IL-6 during course 1 or course 4 (Figures 4C–D and data not shown).

## Discussion

The immunotherapy regimen of ch14.18, GM-CSF and IL-2, has been shown to be efficacious in high-risk NB patients with minimal residual disease and is now the standard FDA-approved therapy (1). This confirmatory study was conducted to collect the additional detailed safety data required to support a BLA to FDA for ch14.18 (dinutuximab), which was successful. The nominal dose of dinutuximab (17.5 mg/m^2^/day) differs from the dose of NCI ch14.18 (25 mg/m^2^) because of a difference in the extinction coefficient used to determine the protein concentration during the manufacturing process, but despite the change in nominal dosing, the amount of antibody delivered per dose is equivalent for the two products. Marachelian et al. reported a dinutuximab dose of 17.5 mg/m^2^ and NCI ch14.18 dose of 25 mg/m^2^ producing comparable exposures, with no notable safety or tolerability differences in a randomized, two-sequence crossover study (17). Overall, this study confirmed the toxicity data and efficacy previously obtained in the ANBL0032 trial (1). Moreover, we demonstrate toxicities that are severe but manageable. No new or unexpected toxicities were encountered.

The side-effect profile of this immunotherapy differs greatly from traditional cytotoxic therapy. While the majority of ch14.18 related toxicities are acute, temporarily related to infusion of antibody, and manageable with supportive care (e.g., fever, neuropathic pain); some can be life threatening and impact the ability to administer this therapy (e.g., anaphylaxis and/or bronchospasm). This study confirmed the need for close patient monitoring with hospitalization for administration of the described administration schedule to ensure timely response to required supportive care. Infrequently, ocular toxicities of diplopia, dilated pupils or both occurred, lasting for longer duration.

We chose to prolong the initial infusion time of ch14.18 to ≥10 h for all patients in this study compared to the 5.75 h infusion on ANBL0032. There is evidence that prolongation of the infusion time is associated with less toxicity. Lode et al. has reported decreased toxicity with continuous IV infusion of ch14.18/CHO (ch14.18 produced in Chinese hamster ovary cells) over 10 days in combination with IL-2 when compared to the same dose of ch14.18/CHO (100 mg/m^2^ as total dose) administered IV daily over 8 h for 5 days (18). This prolonged continuous infusion approach allowed a comparable dose of ch14.18/CHO to that used here, to be administered in the outpatient setting. Analysis of the safety and toxicity associated with short *vs*. prolonged (≥10 h) infusion of ch14.18 on ANBL0032 is planned and when combined with the results of this and other studies will help determine the optimal infusion duration that may mitigate toxicities and potentially the cost of therapy administration.

Navid et al. reported the results from a phase I trial using a novel anti-GD2 agent, hu14.18K322A, designed to decrease neuropathic toxicity through decreased complement binding (4). Kushner et al. reported multifold dose escalation of heat modified 3F8 resulting in less toxicity and allowing administration in the outpatient setting (19). However, the differences in administration schedule, cytokine use and toxicity grading impair our ability to directly compare toxicity rates across these clinical trials (20).

The pharmacokinetics of ch14.18 in children with NB was previously characterized by intra- and inter-patient variability (21). Desai et al. studied detailed pharmacokinetics of ch14.18 in 14 pediatric subjects who received identical immunotherapy as delivered on ANBL0931 and demonstrated less variable ch14.18 disposition than previously reported (13). In comparison, pharmacokinetics profiles obtained through a very limited sampling on ANBL0931 demonstrated lower mean peak ch14.18 concentrations (5.5 vs 11 mcg/ml) but higher mean trough concentrations (0.78 vs 0.2 mcg/ml) than reported by Desai. This difference could be a function of limited sampling in both studies. Similar to prior experience with ch14.18 when administered following ASCT in patients with NB (1), the development of HACA was uncommon. No dose modification was done based on HACA results in our patients.

Cytokines can orchestrate inflammation and immunity and can be key players in toxicity reactions such as AR in general, and anaphylaxis in particular. The course-associated average increases in cytokine levels reflected individual increases in the vast majority of patients despite a large range of variation. These increases may be due to the administration of cytokines GM-CSF and IL-2 during therapy, due to ch14.18, or both. However, cytokine levels of the patients experiencing AR appeared similar to those of patients who did not experience AR. IL-5 exhibited the largest course-associated fold-increase in patients vs. normal controls, as much as 20,000-fold higher and is reported to promote IgE and eosinophilic responses (22), while IL-5 did have an association with hypotension, it did not have an association with AR in patients on immunotherapy.

Cytokines can also promote tumor cell growth, migration, and metastasis or create an immunosuppressive microenvironment (23). CXCL9 is a T- and NK-cell chemoattractant that displays antitumor and pro-tumor activities. Consistent with this observation, high levels of serum CL1 chemokine cluster, which includes CXCL9, was correlated with an shorter OS in chronic lymphocytic leukemia (11), suggesting that high levels of CXCL9 and related chemokines might predict a poor prognosis in NB as well.

IL-6 has also been reported to be part of a multi-protein biomarkers signature in a retrospective study to be associated with relapse in high risk NB (24). In this study, there was no association of IL-6 with event or outcome before and during maintenance phase immunotherapy plus cytokines. However, we must caution about possible variable time between blood and plasma isolation in the reported retrospective study (24), knowing that handling of sample can greatly affect measured levels of cytokines (25, 26), and our own data demonstrating highly variable and often unreliable cytokine values when measured in plasma (unpublished observations). Thus, while IL-6 levels may be able to predict outcome for some types of chemotherapy when measured at diagnosis, or retrospectively predict relapse, its level at the start of maintenance phase with ch14.18 immunotherapy is not prognostic of outcome.

The induction of nitric oxide as a consequence of IL-2 administration is thought to be responsible for immunotherapy-associated capillary leak syndrome and hypotension (7, 8). However, nitric oxide levels, as determined through the measurement of the more stable metabolite nitrate, were slightly but significantly *decreased* in a paired samples analysis during both GM-CSF and IL-2 courses of therapy, and were paradoxically associated with severe hypotension in the non-IL-2 containing course 1. In the single patient who developed course 1 severe capillary leak, nitrate levels were similar to those of the group as a whole. These data suggest that the cause of immunotherapy-associated vascular disorders is not due to nitrate release. However, given the known role of nitric oxide in vascular relaxation, it is hard to understand the decrease in nitrate levels during immunotherapy, or why lower levels might be associated with hypotension, though it may be as simple as timing of the blood sample draw versus the observation of toxicity.

Several cytokines showed associations with the occurrence of hypotension and capillary leak. However, as with nitrate, lower levels of some cytokines were associated with vascular disorders. In particular, the capillary leak patients had some of the *lowest* cytokine levels of all samples analyzed. Considering the dramatic increase in cytokines we have observed during the course of therapy and the reported relationships of elevated TNFα and IL-6 with vascular permeability (27), this is a paradoxical observation. One might speculate that the increase in cytokines we observed during therapy may be a protective response that helps maintain vascular integrity. In patients with low cytokine levels, this protective response may be compromised and be reflected in the observed vascular disorders. Thus, when compared to patients whose cytokine levels remain elevated, these patients would appear to have very low cytokine levels. Finally, the possibility that immunotherapy is suppressing cytokine release in some patients cannot be excluded.

In summary, this study confirmed the significant, but manageable treatment-related toxicities of the immunotherapy including pain, ARs, hypotension and capillary leak syndrome observed in the ANBL0032 study, patterned the dramatic increase in cytokine release during immunotherapy, validated the excellent EFS and OS rates of this immunotherapy combination treatment (1) and implicated the chemokine CXCL9 as mildly prognostic at pretreatment.

## Ethics Statement

Written informed consent was obtained from parents or legal guardians. Patients were treated at thirty Children’s Oncology Group institutions on a protocol approved by the institution’s local Institutional Review Board (IRB) or National Cancer Institute (NCI)-sponsored pediatric central institutional review board (NCT01041638; see Appendix for the list of institutions, online only).

## Author Contributions

Conception and design: PS, AY, WL, JM, MO, AG, RS, CR, JP, and MS. Development of methodology: PS, AY, MO, AG, WL, JM, RS, CR, MS, JP. Acquisition of data: MO, AG, AY, MD, WL, AN, ST, BS, MP, LS, JM, JP. Analysis and interpretation of data (e.g., statistical analysis, biostatistics, computational analysis): AN, ST, WL, MO, AG, AY, MD, KM, PS, LS, MS. Writing, review and/or revision of the manuscript: MO, AN, WL, ST, AG, PS, AY, MD, LS, JP, and MS. Administrative, technical or material support (i.e., reporting or organizing data, constructing databases): AN, ST, WL, MO, AG, AY, MD, PS, LS, JP. Study supervision: MO, AG, AY, PS, JP, JM, MS.

## Conflict of Interest Statement

The authors declare that the research was conducted in the absence of any commercial or financial relationships that could be construed as a potential conflict of interest. The reviewers, JF and CL, and the handling Editor declared their shared affiliation.
