# Corrigendum: A Comprehensive Safety Trial of Chimeric Antibody 14.18 With GM-CSF, IL-2, and Isotretinoin in High-Risk Neuroblastoma Patients Following Myeloablative Therapy: Children’s Oncology Group Study ANBL0931

**DOI:** 10.3389/fimmu.2018.01641

**Published:** 2018-07-16

**Authors:** M. Fevzi Ozkaynak, Andrew L. Gilman, Wendy B. London, Arlene Naranjo, Mitchell B. Diccianni, Sheena C. Tenney, Malcolm Smith, Karen S. Messer, Robert Seeger, C. Patrick Reynolds, L. Mary Smith, Barry L. Shulkin, Marguerite Parisi, John M. Maris, Julie R. Park, Paul M. Sondel, Alice L. Yu

**Affiliations:** ^1^New York Medical College, Valhalla, NY, United States; ^2^Levine Children’s Hospital, Charlotte, NC, United States; ^3^Dana Farber Cancer Institute and Boston Children’s Hospital, Harvard Medical School, Boston, MA, United States; ^4^Children’s Oncology Group Statistics and Data Center, University of Florida, Gainesville, FL, United States; ^5^Moores Cancer Center, University of California, San Diego, La Jolla, CA, United States; ^6^National Cancer Institute, Bethesda, MD, United States; ^7^Children’s Hospital Los Angeles, University Southern California, Los Angeles, Los Angeles, CA, United States; ^8^Texas Tech University Health Sciences Center, Lubbock, TX, United States; ^9^United Therapeutics, Silver Spring, MD, United States; ^10^St. Jude’s Children’s Research Hospital, Memphis, TN, United States; ^11^Seattle Children’s Hospital, University of Washington School of Medicine, Seattle, WA, United States; ^12^Children’s Hospital of Philadelphia, Philadelphia, PA, United States; ^13^University of Wisconsin Carbone Cancer Center, Madison, WI, United States; ^14^Institute of Stem Cell and Translational Cancer Research, Chang Gung Memorial Hospital, Taoyuan, Taiwan

**Keywords:** neuroblastoma, immunotherapy, anti-GD2 chimeric antibody, cytokines, safety, cytokine biomarkers

In the original article, we neglected to include the funder NCTN Operations Center Grant U10CA180886 (current grant, as of 3/1/14) St. Baldrick’s Foundation.

The revised “Funding” should read:

This work was supported by ARRA grant from the National Institutes of Health, by grant from the Department of Defense (AY) # W81XWH-12-1-0427, by grant from the NIH (AY) #5R01CA164132, the Lou Bridgeman Memorial Fund, the Alex’s Lemonade Stand Foundation (AY), NCTN Operations Center Grant U10CA180886 (current grant, as of 3/1/14) St. Baldrick’s Foundation and COG SDC grant U10CA180899.

The authors apologize for this error and state that this does not change the scientific conclusions of the article in any way.

The original article has been updated.

## Conflict of Interest Statement

The authors declare that the research was conducted in the absence of any commercial or financial relationships that could be construed as a potential conflict of interest.

