# Supplementary material for: A Comprehensive Safety Trial of Chimeric Antibody 14.18 With GM-CSF, IL-2, and Isotretinoin in High-Risk Neuroblastoma Patients Following Myeloablative Therapy: Children’s Oncology Group Study ANBL0931
Source: Front Immunol. 2018 Jun 18;9:1355. doi: 10.3389/fimmu.2018.01355 (PMC6016521; doi:10.3389/fimmu.2018.01355)
Supplement: Supplementary file 1 [file table_1.PDF]

SUPPLEMENTAL TABLE: Immunotherapy ch14.18 + GM-CSF + IL-2+ Isotretinoin

ANBL0931 Reporting Period 1: Immunotherapy ch14.18 + GM-CSF + Isotretinoin

Non-Targeted Toxicities		Grade 1 or 2 (N = 104)	
		count	Incidence (%)
**Primary Category**			
**Blood and lymphatic system disorders**	**13200-Anemia**	85	***81.7***
	**17200-Blood and lymphatic system disorders - Other specify**	1	***1.0***
	**25800-Disseminated intravascular coagulation**	1	***1.0***
**Cardiac disorders**	**20100-Cardiac disorders - Other specify**	3	***2.9***
	**21300-Chest pain - cardiac**	2	***1.9***
	**63100-Pericardial effusion**	1	***1.0***
	**74200-Sinus bradycardia**	4	***3.8***
	**74500-Sinus tachycardia**	64	***61.5***
	**78900-Supraventricular tachycardia**	1	***1.0***
	**81400-Tricuspid valve disease**	1	***1.0***
**Ear and labyrinth disorders**	**28000-Ear pain**	1	***1.0***
	**31600-External ear pain**	1	***1.0***
**Endocrine disorders**	**41900-Hyperparathyroidism**	1	***1.0***
	**43800-Hypothyroidism**	1	***1.0***
**Eye disorders**	**17800-Blurred vision**	2	***1.9***
	**26000-Dry eye**	1	***1.0***
	**31900-Eye disorders - Other specify**	6	***5.8***
	**32100-Eye pain**	1	***1.0***
	**59300-Optic nerve disorder**	1	***1.0***

SUPPLEMENTAL TABLE: Immunotherapy ch14.18 + GM-CSF + IL-2+ Isotretinoin

ANBL0931 Reporting Period 1: Immunotherapy ch14.18 + GM-CSF + Isotretinoin

Non-Targeted Toxicities		Grade 1 or 2 (*N* = 104)	
		count	Incidence (%)
**Gastrointestinal disorders**	**10100-Abdominal distension**	3	***2.9***
	**10300-Abdominal pain**	46	***44.2***
	**12800-Anal pain**	1	***1.0***
	**21200-Cheilitis**	7	***6.7***
	**23400-Constipation**	14	***13.5***
	**25700-Diarrhea**	28	***26.9***
	**29400-Enterocolitis**	3	***2.9***
	**34400-Flatulence**	2	***1.9***
	**36900-Gastrointestinal pain**	1	***1.0***
	**57600-Nausea**	38	***36.5***
	**59700-Oral pain**	1	***1.0***
	**77900-Stomach pain**	2	***1.9***
	**87900-Vomiting**	42	***40.4***

SUPPLEMENTAL TABLE: Immunotherapy ch14.18 + GM-CSF + IL-2+ Isotretinoin

ANBL0931 Reporting Period 1: Immunotherapy ch14.18 + GM-CSF + Isotretinoin

Non-Targeted Toxicities		Grade 1 or 2 (*N* = 104) count	Grade 1 or 2 (*N* = 104) Incidence (%)
General disorders and administration site conditions	21500-Chills	11	*10.6*
	28200-Edema face	18	*17.3*
	28300-Edema limbs	11	*10.6*
	28400-Edema trunk	1	*1.0*
	32500-Facial pain	1	*1.0*
	33200-Fatigue	9	*8.7*
	33900-Fever	93	*89.4*
	34600-Flu like symptoms	1	*1.0*
	35000-Gait disturbance	1	*1.0*
	37200-General disorders and administration site conditions - Other specify	1	*1.0*
	43700-Hypothermia	1	*1.0*
	45000-Infusion related reaction	2	*1.9*
	45200-Injection site reaction	2	*1.9*
	48700-Irritability	14	*13.5*
	52800-Localized edema	4	*3.8*
	54000-Malaise	1	*1.0*
	58600-Non-cardiac chest pain	2	*1.9*
	60600-Pain	55	*52.9*
Immune system disorders	12000-Allergic reaction	19	*18.3*
Infections and infestations	20500-Catheter related infection	1	*1.0*
	29500-Enterocolitis infectious	2	*1.9*
	30400-Esophageal infection	1	*1.0*
	44800-Infections and infestations - Other specify	5	*4.8*
	57200-Nail infection	1	*1.0*
	60100-Otitis media	1	*1.0*
	72600-Rhinitis infective	2	*1.9*
	75200-Skin infection	1	*1.0*
	82300-Upper respiratory infection	1	*1.0*
	83100-Urinary tract infection	1	*1.0*

SUPPLEMENTAL TABLE: Immunotherapy ch14.18 + GM-CSF + IL-2+ Isotretinoin

ANBL0931 Reporting Period 1: Immunotherapy ch14.18 + GM-CSF + Isotretinoin

Non-Targeted Toxicities		Grade 1 or 2 (*N* = 104)	
		count	Incidence (%)
**Injury, poisoning and procedural complications**	**19400-Bruising**	4	***3.8***
	**45700-Injury poisoning and procedural complications - Other specify**	1	***1.0***
	**86400-Vascular access complication**	4	***3.8***
**Investigations**	**10900-Activated partial thromboplastin time prolonged**	2	***1.9***
	**11600-Alanine aminotransferase increased**	65	***62.5***
	**11800-Alkaline phosphatase increased**	7	***6.7***
	**15000-Aspartate aminotransferase increased**	54	***51.9***
	**17400-Blood bilirubin increased**	5	***4.8***
	**21700-Cholesterol high**	1	***1.0***
	**24100-Creatinine increased**	11	***10.6***
	**37500-GGT increased**	13	***12.5***
	**39400-Hemoglobin increased**	3	***2.9***
	**53700-Lymphocyte count decreased**	76	***73.1***
	**58300-Neutrophil count decreased**	22	***21.2***
	**65800-Platelet count decreased**	71	***68.3***
	**88200-Weight gain**	23	***22.1***
	**88300-Weight loss**	6	***5.8***
	**88500-White blood cell decreased**	68	***65.4***

SUPPLEMENTAL TABLE: Immunotherapy ch14.18 + GM-CSF + IL-2+ Isotretinoin

ANBL0931 Reporting Period 1: Immunotherapy ch14.18 + GM-CSF + Isotretinoin

Non-Targeted Toxicities		Grade 1 or 2 (*N* = 104)	
		count	Incidence (%)
**Metabolism and nutrition disorders**	**10700-Acidosis**	2	***1.9***
	**13500-Anorexia**	17	***16.3***
	**24700-Dehydration**	2	***1.9***
	**41300-Hypercalcemia**	8	***7.7***
	**41400-Hyperglycemia**	27	***26.0***
	**41600-Hyperkalemia**	7	***6.7***
	**41700-Hypermagnesemia**	6	***5.8***
	**41800-Hypernatremia**	3	***2.9***
	**42400-Hypertriglyceridemia**	51	***49.0***
	**42600-Hypoalbuminemia**	92	***88.5***
	**42700-Hypocalcemia**	71	***68.3***
	**42900-Hypoglycemia**	14	***13.5***
	**43100-Hypokalemia**	55	***52.9***
	**43200-Hypomagnesemia**	22	***21.2***
	**43300-Hyponatremia**	71	***68.3***
	**43500-Hypophosphatemia**	26	***25.0***
	**54900-Metabolism and nutrition disorders - Other specify**	2	***1.9***
**Musculoskeletal and connective tissue disorders**	**14700-Arthralgia**	2	***1.9***
	**16200-Back pain**	11	***10.6***
	**18200-Bone pain**	2	***1.9***
	**19700-Buttock pain**	1	***1.0***
	**21400-Chest wall pain**	3	***2.9***
	**37300-Generalized muscle weakness**	1	***1.0***
	**57800-Neck pain**	4	***3.8***
	**60700-Pain in extremity**	22	***21.2***

SUPPLEMENTAL TABLE: Immunotherapy ch14.18 + GM-CSF + IL-2+ Isotretinoin

ANBL0931 Reporting Period 1: Immunotherapy ch14.18 + GM-CSF + Isotretinoin

Non-Targeted Toxicities		Grade 1 or 2 (*N* = 104) count	Incidence (%)
Nervous system disorders	11500-Akathisia	1	*1.0*
	22000-Cognitive disturbance	1	*1.0*
	38800-Headache	9	*8.7*
	55400-Movements involuntary	1	*1.0*
	58200-Neuralgia	3	*2.9*
	62100-Paresthesia	2	*1.9*
	64100-Peripheral sensory neuropathy	2	*1.9*
	76700-Somnolence	2	*1.9*
	81300-Tremor	1	*1.0*
Psychiatric disorders	11400-Agitation	7	*6.7*
	13700-Anxiety	4	*3.8*
	23000-Confusion	1	*1.0*
	25400-Depression	2	*1.9*
	38500-Hallucinations	3	*2.9*
	45900-Insomnia	3	*2.9*
	68500-Psychiatric disorders - Other specify	2	*1.9*
Renal and urinary disorders	39300-Hematuria	13	*12.5*
	68300-Proteinuria	19	*18.3*
	71000-Renal and urinary disorders - Other specify	1	*1.0*
	83000-Urinary retention	17	*16.3*
	83300-Urinary tract pain	3	*2.9*
	83500-Urine discoloration	2	*1.9*
Reproductive system and breast disorders	62900-Penile pain	1	*1.0*

SUPPLEMENTAL TABLE: Immunotherapy ch14.18 + GM-CSF + IL-2+ Isotretinoin

ANBL0931 Reporting Period 1: Immunotherapy ch14.18 + GM-CSF + Isotretinoin

Non-Targeted Toxicities		Grade 1 or 2 (*N* = 104)	
		count	Incidence (%)
**Respiratory, thoracic and mediastinal disorders**	**12100-Allergic rhinitis**	6	***5.8***
	**15400-Atelectasis**	2	***1.9***
	**19000-Bronchial stricture**	1	***1.0***
	**19300-Bronchospasm**	5	***4.8***
	**23800-Cough**	30	***28.8***
	**27800-Dyspnea**	1	***1.0***
	**29700-Epistaxis**	2	***1.9***
	**41000-Hoarseness**	1	***1.0***
	**43900-Hypoxia**	12	***11.5***
	**57500-Nasal congestion**	5	***4.8***
	**65900-Pleural effusion**	1	***1.0***
	**66300-Pneumonitis**	1	***1.0***
	**66700-Postnasal drip**	1	***1.0***
	**67600-Productive cough**	1	***1.0***
	**71600-Respiratory thoracic and mediastinal disorders - Other specify**	18	***17.3***
	**76200-Sneezing**	3	***2.9***
	**76800-Sore throat**	1	***1.0***
	**88400-Wheezing**	3	***2.9***

SUPPLEMENTAL TABLE: Immunotherapy ch14.18 + GM-CSF + IL-2+ Isotretinoin

ANBL0931 Reporting Period 1: Immunotherapy ch14.18 + GM-CSF + Isotretinoin

Non-Targeted Toxicities		Grade 1 or 2 (*N* = 104)	
		count	Incidence (%)
**Skin and subcutaneous tissue disorders**	**12200-Alopecia**	10	***9.6***
	**19500-Bullous dermatitis**	1	***1.0***
	**26200-Dry skin**	27	***26.0***
	**29900-Erythema multiforme**	2	***1.9***
	**42300-Hypertrichosis**	1	***1.0***
	**63600-Periorbital edema**	4	***3.8***
	**68400-Pruritus**	38	***36.5***
	**69600-Rash acneiform**	2	***1.9***
	**69700-Rash maculo-papular**	21	***20.2***
	**74700-Skin and subcutaneous tissue disorders - Other specify**	6	***5.8***
	**74900-Skin hyperpigmentation**	1	***1.0***
	**75000-Skin hypopigmentation**	2	***1.9***
	**75100-Skin induration**	1	***1.0***
	**75300-Skin ulceration**	1	***1.0***
	**84100-Urticaria**	17	***16.3***
**Vascular disorders**	**19800-Capillary leak syndrome**	24	***23.1***
	**34700-Flushing**	2	***1.9***
	**41100-Hot flashes**	2	***1.9***
	**42100-Hypertension**	29	***27.9***
	**43600-Hypotension**	58	***55.8***
	**79600-Thromboembolic event**	1	***1.0***
	**86500-Vascular disorders - Other specify**	1	***1.0***

ANBL0931 Reporting Period 2: Immunotherapy ch14.18 + IL-2 + Isotretinoin

Non-Targeted Toxicities		Grade 1 or 2 (*N* = 100)	
		count	Incidence (%)
**Primary Category**			
**Blood and lymphatic system disorders**	**13200-Anemia**	85	***85.0***
**Cardiac disorders**	**61000-Palpitations**	2	***2.0***
	**74200-Sinus bradycardia**	2	***2.0***
	**74500-Sinus tachycardia**	64	***64.0***
	**78900-Supraventricular tachycardia**	1	***1.0***
**Ear and labyrinth disorders**	**27900-Ear and labyrinth disorders - Other specify**	1	***1.0***
**Endocrine disorders**	**42200-Hyperthyroidism**	1	***1.0***
**Eye disorders**	**17800-Blurred vision**	3	***3.0***
	**31900-Eye disorders - Other specify**	4	***4.0***
	**32100-Eye pain**	4	***4.0***
**Gastrointestinal disorders**	**10100-Abdominal distension**	9	***9.0***
	**10300-Abdominal pain**	32	***32.0***
	**12800-Anal pain**	1	***1.0***
	**17100-Bloating**	1	***1.0***
	**21200-Cheilitis**	4	***4.0***
	**23400-Constipation**	14	***14.0***
	**25700-Diarrhea**	34	***34.0***
	**27500-Dyspepsia**	1	***1.0***
	**27600-Dysphagia**	2	***2.0***
	**29400-Enterocolitis**	1	***1.0***
	**36400-Gastritis**	1	***1.0***
	**36500-Gastroesophageal reflux disease**	1	***1.0***
	**36700-Gastrointestinal disorders - Other specify**	2	***2.0***
	**37600-Gingival pain**	1	***1.0***
	**39800-Hemorrhoidal hemorrhage**	1	***1.0***
	**57600-Nausea**	33	***33.0***
	**59700-Oral pain**	3	***3.0***
	**77900-Stomach pain**	2	***2.0***
	**80200-Toothache**	1	***1.0***
	**87900-Vomiting**	44	***44.0***

ANBL0931 Reporting Period 2: Immunotherapy ch14.18 + IL-2 + Isotretinoin

Non-Targeted Toxicities		Grade 1 or 2 (*N* = 100)	
		count	Incidence (%)
General disorders and administration site conditions	21500-Chills	8	*8.0*
	28200-Edema face	32	*32.0*
	28300-Edema limbs	21	*21.0*
	28400-Edema trunk	3	*3.0*
	33200-Fatigue	18	*18.0*
	33900-Fever	89	*89.0*
	34600-Flu like symptoms	2	*2.0*
	35000-Gait disturbance	2	*2.0*
	37200-General disorders and administration site conditions - Other specify	1	*1.0*
	45000-Infusion related reaction	1	*1.0*
	48700-Irritability	16	*16.0*
	52800-Localized edema	2	*2.0*
	54000-Malaise	2	*2.0*
	60600-Pain	50	*50.0*
Immune system disorders	12000-Allergic reaction	21	*21.0*
Infections and infestations	20500-Catheter related infection	2	*2.0*
	29500-Enterocolitis infectious	2	*2.0*
	44800-Infections and infestations - Other specify	3	*3.0*
	62200-Paronychia	1	*1.0*
	65200-Pharyngitis	1	*1.0*
	74600-Sinusitis	1	*1.0*
	75200-Skin infection	2	*2.0*
	76000-Small intestine infection	1	*1.0*
	82300-Upper respiratory infection	3	*3.0*
	83100-Urinary tract infection	2	*2.0*
Injury, poisoning and procedural complications	45700-Injury poisoning and procedural complications - Other specify	1	*1.0*
	86400-Vascular access complication	2	*2.0*

ANBL0931 Reporting Period 2: Immunotherapy ch14.18 + IL-2 + Isotretinoin

Non-Targeted Toxicities		Grade 1 or 2 (*N* = 100)	
		count	Incidence (%)
**Investigations**	**10900-Activated partial thromboplastin time prolonged**	1	***1.0***
	**11600-Alanine aminotransferase increased**	64	***64.0***
	**11800-Alkaline phosphatase increased**	9	***9.0***
	**15000-Aspartate aminotransferase increased**	72	***72.0***
	**17400-Blood bilirubin increased**	10	***10.0***
	**24100-Creatinine increased**	15	***15.0***
	**37500-GGT increased**	8	***8.0***
	**39400-Hemoglobin increased**	5	***5.0***
	**45800-INR increased**	2	***2.0***
	**48400-Investigations - Other specify**	1	***1.0***
	**53700-Lymphocyte count decreased**	80	***80.0***
	**53800-Lymphocyte count increased**	1	***1.0***
	**58300-Neutrophil count decreased**	60	***60.0***
	**65800-Platelet count decreased**	79	***79.0***
	**88200-Weight gain**	34	***34.0***
	**88300-Weight loss**	10	***10.0***
	**88500-White blood cell decreased**	74	***74.0***

ANBL0931 Reporting Period 2: Immunotherapy ch14.18 + IL-2 + Isotretinoin

Non-Targeted Toxicities		Grade 1 or 2 (*N* = 100)	
		count	Incidence (%)
**Metabolism and nutrition disorders**	**10700-Acidosis**	1	***1.0***
	**13500-Anorexia**	17	***17.0***
	**24700-Dehydration**	3	***3.0***
	**41300-Hypercalcemia**	10	***10.0***
	**41400-Hyperglycemia**	36	***36.0***
	**41600-Hyperkalemia**	11	***11.0***
	**41700-Hypermagnesemia**	14	***14.0***
	**41800-Hypernatremia**	4	***4.0***
	**42400-Hypertriglyceridemia**	32	***32.0***
	**42600-Hypoalbuminemia**	89	***89.0***
	**42700-Hypocalcemia**	77	***77.0***
	**42900-Hypoglycemia**	21	***21.0***
	**43100-Hypokalemia**	66	***66.0***
	**43200-Hypomagnesemia**	20	***20.0***
	**43300-Hyponatremia**	78	***78.0***
	**43500-Hypophosphatemia**	52	***52.0***
	**54900-Metabolism and nutrition disorders - Other specify**	1	***1.0***
**Musculoskeletal and connective tissue disorders**	**16200-Back pain**	11	***11.0***
	**19700-Buttock pain**	1	***1.0***
	**21400-Chest wall pain**	1	***1.0***
	**55900-Muscle weakness lower limb**	1	***1.0***
	**56300-Musculoskeletal and connective tissue disorder - Other specify**	2	***2.0***
	**56500-Myalgia**	4	***4.0***
	**57800-Neck pain**	7	***7.0***
	**60700-Pain in extremity**	22	***22.0***

ANBL0931 Reporting Period 2: Immunotherapy ch14.18 + IL-2 + Isotretinoin

Non-Targeted Toxicities		Grade 1 or 2 (*N* = 100)	
		count	Incidence (%)
**Nervous system disorders**	**25300-Depressed level of consciousness**	1	***1.0***
	**25900-Dizziness**	2	***2.0***
	**38800-Headache**	11	***11.0***
	**51800-Lethargy**	2	***2.0***
	**54400-Memory impairment**	1	***1.0***
	**58200-Neuralgia**	2	***2.0***
	**64100-Peripheral sensory neuropathy**	2	***2.0***
	**73600-Seizure**	2	***2.0***
	**76700-Somnolence**	2	***2.0***
**Psychiatric disorders**	**11400-Agitation**	11	***11.0***
	**13700-Anxiety**	3	***3.0***
	**23000-Confusion**	5	***5.0***
	**38500-Hallucinations**	8	***8.0***
	**45900-Insomnia**	4	***4.0***
	**68500-Psychiatric disorders - Other specify**	1	***1.0***
	**71700-Restlessness**	2	***2.0***
**Renal and urinary disorders**	**11100-Acute kidney injury**	1	***1.0***
	**21800-Chronic kidney disease**	1	***1.0***
	**39300-Hematuria**	15	***15.0***
	**68300-Proteinuria**	26	***26.0***
	**82800-Urinary frequency**	1	***1.0***
	**83000-Urinary retention**	10	***10.0***
	**83300-Urinary tract pain**	1	***1.0***
	**83500-Urine discoloration**	2	***2.0***
**Reproductive system and breast disorders**	**37400-Genital edema**	1	***1.0***
	**62600-Pelvic pain**	1	***1.0***

ANBL0931 Reporting Period 2: Immunotherapy ch14.18 + IL-2 + Isotretinoin

Non-Targeted Toxicities		Grade 1 or 2 (*N* = 100) count	Grade 1 or 2 (*N* = 100) Incidence (%)
Respiratory, thoracic and mediastinal disorders	12100-Allergic rhinitis	3	***3.0***
	15400-Atelectasis	3	***3.0***
	19300-Bronchospasm	2	***2.0***
	23800-Cough	37	***37.0***
	27800-Dyspnea	3	***3.0***
	41000-Hoarseness	1	***1.0***
	43900-Hypoxia	21	***21.0***
	57500-Nasal congestion	5	***5.0***
	65300-Pharyngolaryngeal pain	1	***1.0***
	65900-Pleural effusion	1	***1.0***
	67600-Productive cough	1	***1.0***
	68700-Pulmonary edema	2	***2.0***
	71600-Respiratory thoracic and mediastinal disorders - Other specify	19	***19.0***
	72200-Retinoic acid syndrome	1	***1.0***
	76800-Sore throat	2	***2.0***
	88400-Wheezing	5	***5.0***
Skin and subcutaneous tissue disorders	12200-Alopecia	3	***3.0***
	26200-Dry skin	46	***46.0***
	29900-Erythema multiforme	4	***4.0***
	57300-Nail loss	1	***1.0***
	63600-Periorbital edema	5	***5.0***
	68400-Pruritus	29	***29.0***
	69600-Rash acneiform	1	***1.0***
	69700-Rash maculo-papular	24	***24.0***
	74700-Skin and subcutaneous tissue disorders - Other specify	5	***5.0***
	74800-Skin atrophy	1	***1.0***
	75000-Skin hypopigmentation	1	***1.0***
	84100-Urticaria	25	***25.0***

ANBL0931 Reporting Period 2: Immunotherapy ch14.18 + IL-2 + Isotretinoin

Non-Targeted Toxicities		Grade 1 or 2 (*N* = 100)	
		count	Incidence (%)
**Vascular disorders**	**19800-Capillary leak syndrome**	36	***36.0***
	**34700-Flushing**	5	***5.0***
	**42100-Hypertension**	40	***40.0***
	**43600-Hypotension**	57	***57.0***
	**86500-Vascular disorders - Other specify**	1	***1.0***

ANBL0931 Reporting Period 3: Immunotherapy ch14.18 + GM-CSF + Isotretinoin

Non-Targeted Toxicities		Grade 1 or 2 (*N* = 98)	
		count	Incidence (%)
**Primary Category**			
**Blood and lymphatic system disorders**	**13200-Anemia**	80	***81.6***
	**17200-Blood and lymphatic system disorders - Other specify**	1	***1.0***
**Cardiac disorders**	**20100-Cardiac disorders - Other specify**	2	***2.0***
	**63100-Pericardial effusion**	1	***1.0***
	**74200-Sinus bradycardia**	1	***1.0***
	**74500-Sinus tachycardia**	56	***57.1***
	**78900-Supraventricular tachycardia**	1	***1.0***
**Eye disorders**	**17800-Blurred vision**	1	***1.0***
	**31900-Eye disorders - Other specify**	1	***1.0***
	**59300-Optic nerve disorder**	1	***1.0***
	**65600-Photophobia**	2	***2.0***
	**73200-Scleral disorder**	1	***1.0***
**Gastrointestinal disorders**	**10100-Abdominal distension**	4	***4.1***
	**10300-Abdominal pain**	28	***28.6***
	**21200-Cheilitis**	2	***2.0***
	**22100-Colitis**	1	***1.0***
	**23400-Constipation**	12	***12.2***
	**25700-Diarrhea**	18	***18.4***
	**26100-Dry mouth**	1	***1.0***
	**34400-Flatulence**	1	***1.0***
	**36500-Gastroesophageal reflux disease**	1	***1.0***
	**36700-Gastrointestinal disorders - Other specify**	1	***1.0***
	**57600-Nausea**	18	***18.4***
	**70800-Rectal ulcer**	1	***1.0***
	**77900-Stomach pain**	1	***1.0***
	**87900-Vomiting**	21	***21.4***

ANBL0931 Reporting Period 3: Immunotherapy ch14.18 + GM-CSF + Isotretinoin

Non-Targeted Toxicities		Grade 1 or 2 (*N* = 98)	
		count	Incidence (%)
General disorders and administration site conditions	21500-Chills	2	***2.0***
	28200-Edema face	16	***16.3***
	28300-Edema limbs	7	***7.1***
	28400-Edema trunk	1	***1.0***
	32500-Facial pain	1	***1.0***
	33200-Fatigue	10	***10.2***
	33900-Fever	68	***69.4***
	34600-Flu like symptoms	1	***1.0***
	35000-Gait disturbance	1	***1.0***
	37200-General disorders and administration site conditions - Other specify	1	***1.0***
	45000-Infusion related reaction	1	***1.0***
	45200-Injection site reaction	6	***6.1***
	48700-Irritability	8	***8.2***
	52800-Localized edema	2	***2.0***
	58600-Non-cardiac chest pain	2	***2.0***
	60600-Pain	48	***49.0***
Immune system disorders	12000-Allergic reaction	10	***10.2***
Infections and infestations	18800-Bronchial infection	1	***1.0***
	29500-Enterocolitis infectious	3	***3.1***
	44800-Infections and infestations - Other specify	1	***1.0***
	53100-Lung infection	2	***2.0***
	65200-Pharyngitis	1	***1.0***
	72600-Rhinitis infective	1	***1.0***
	82300-Upper respiratory infection	1	***1.0***
Injury, poisoning and procedural complications	19400-Bruising	2	***2.0***
	86400-Vascular access complication	1	***1.0***

ANBL0931 Reporting Period 3: Immunotherapy ch14.18 + GM-CSF + Isotretinoin

Non-Targeted Toxicities		Grade 1 or 2 (*N* = 98)	
		count	Incidence (%)
**Investigations**	**10900-Activated partial thromboplastin time prolonged**	1	***1.0***
	**11600-Alanine aminotransferase increased**	44	***44.9***
	**11800-Alkaline phosphatase increased**	6	***6.1***
	**15000-Aspartate aminotransferase increased**	36	***36.7***
	**17400-Blood bilirubin increased**	3	***3.1***
	**21700-Cholesterol high**	1	***1.0***
	**24100-Creatinine increased**	11	***11.2***
	**37500-GGT increased**	8	***8.2***
	**39400-Hemoglobin increased**	3	***3.1***
	**48400-Investigations - Other specify**	1	***1.0***
	**53700-Lymphocyte count decreased**	57	***58.2***
	**53800-Lymphocyte count increased**	1	***1.0***
	**58300-Neutrophil count decreased**	17	***17.3***
	**65800-Platelet count decreased**	55	***56.1***
	**88200-Weight gain**	25	***25.5***
	**88300-Weight loss**	8	***8.2***
	**88500-White blood cell decreased**	41	***41.8***

ANBL0931 Reporting Period 3: Immunotherapy ch14.18 + GM-CSF + Isotretinoin

Non-Targeted Toxicities		Grade 1 or 2 (*N* = 98)	
		count	Incidence (%)
Metabolism and nutrition disorders	10700-Acidosis	1	*1.0*
	13500-Anorexia	7	*7.1*
	24700-Dehydration	1	*1.0*
	41300-Hypercalcemia	8	*8.2*
	41400-Hyperglycemia	24	*24.5*
	41600-Hyperkalemia	7	*7.1*
	41700-Hypermagnesemia	5	*5.1*
	41800-Hypernatremia	2	*2.0*
	42400-Hypertriglyceridemia	30	*30.6*
	42600-Hypoalbuminemia	73	*74.5*
	42700-Hypocalcemia	52	*53.1*
	42900-Hypoglycemia	9	*9.2*
	43100-Hypokalemia	40	*40.8*
	43200-Hypomagnesemia	12	*12.2*
	43300-Hyponatremia	53	*54.1*
	43500-Hypophosphatemia	13	*13.3*
Musculoskeletal and connective tissue disorders	16200-Back pain	4	*4.1*
	21400-Chest wall pain	1	*1.0*
	56500-Myalgia	4	*4.1*
	57800-Neck pain	6	*6.1*
	60700-Pain in extremity	9	*9.2*
Nervous system disorders	11500-Akathisia	1	*1.0*
	25900-Dizziness	1	*1.0*
	38800-Headache	8	*8.2*
	42000-Hypersomnia	1	*1.0*
	55400-Movements involuntary	1	*1.0*
	58100-Nervous system disorders - Other specify	1	*1.0*
	58200-Neuralgia	3	*3.1*
	64100-Peripheral sensory neuropathy	3	*3.1*
	76700-Somnolence	1	*1.0*

ANBL0931 Reporting Period 3: Immunotherapy ch14.18 + GM-CSF + Isotretinoin

Non-Targeted Toxicities		Grade 1 or 2 (*N* = 98)	
		count	Incidence (%)
Psychiatric disorders	11400-Agitation	9	***9.2***
	13700-Anxiety	5	***5.1***
	23000-Confusion	1	***1.0***
	25400-Depression	2	***2.0***
	38500-Hallucinations	1	***1.0***
	54100-Mania	1	***1.0***
	71700-Restlessness	2	***2.0***
Renal and urinary disorders	39300-Hematuria	12	***12.2***
	68300-Proteinuria	14	***14.3***
	82900-Urinary incontinence	1	***1.0***
	83000-Urinary retention	13	***13.3***
	83300-Urinary tract pain	2	***2.0***
	83400-Urinary urgency	1	***1.0***
	83500-Urine discoloration	2	***2.0***
Reproductive system and breast disorders	37400-Genital edema	1	***1.0***
Respiratory, thoracic and mediastinal disorders	19300-Bronchospasm	3	***3.1***
	23800-Cough	30	***30.6***
	27800-Dyspnea	2	***2.0***
	43900-Hypoxia	8	***8.2***
	57500-Nasal congestion	7	***7.1***
	65900-Pleural effusion	1	***1.0***
	66200-Pleuritic pain	1	***1.0***
	68700-Pulmonary edema	1	***1.0***
	71600-Respiratory thoracic and mediastinal disorders - Other specify	17	***17.3***
	76200-Sneezing	1	***1.0***
	88400-Wheezing	8	***8.2***

ANBL0931 Reporting Period 3: Immunotherapy ch14.18 + GM-CSF + Isotretinoin

Non-Targeted Toxicities		Grade 1 or 2 (*N* = 98)	
		count	Incidence (%)
**Skin and subcutaneous tissue disorders**	**12200-Alopecia**	1	***1.0***
	**26200-Dry skin**	26	***26.5***
	**29900-Erythema multiforme**	2	***2.0***
	**63600-Periorbital edema**	4	***4.1***
	**65700-Photosensitivity**	1	***1.0***
	**68400-Pruritus**	34	***34.7***
	**69700-Rash maculo-papular**	13	***13.3***
	**74700-Skin and subcutaneous tissue disorders - Other specify**	8	***8.2***
	**75000-Skin hypopigmentation**	1	***1.0***
	**75300-Skin ulceration**	2	***2.0***
	**84100-Urticaria**	11	***11.2***
**Vascular disorders**	**19800-Capillary leak syndrome**	14	***14.3***
	**42100-Hypertension**	29	***29.6***
	**43600-Hypotension**	54	***55.1***
	**86500-Vascular disorders - Other specify**	1	***1.0***

ANBL0931 Reporting Period 4: Immunotherapy ch14.18 + IL-2 + Isotretinoin

Non-Targeted Toxicities		Grade 1 or 2 (*N* = 90)	
		count	Incidence (%)
**Primary Category**			
**Blood and lymphatic system disorders**	**13200-Anemia**	79	***87.8***
**Cardiac disorders**	**20100-Cardiac disorders - Other specify**	3	***3.3***
	**21300-Chest pain - cardiac**	1	***1.1***
	**74200-Sinus bradycardia**	4	***4.4***
	**74500-Sinus tachycardia**	56	***62.2***
	**78900-Supraventricular tachycardia**	1	***1.1***
**Ear and labyrinth disorders**	**27900-Ear and labyrinth disorders - Other specify**	1	***1.1***
	**28000-Ear pain**	1	***1.1***
**Endocrine disorders**	**43400-Hypoparathyroidism**	1	***1.1***
**Eye disorders**	**17800-Blurred vision**	1	***1.1***
	**31900-Eye disorders - Other specify**	1	***1.1***
**Gastrointestinal disorders**	**10100-Abdominal distension**	5	***5.6***
	**10300-Abdominal pain**	21	***23.3***
	**21200-Cheilitis**	2	***2.2***
	**23400-Constipation**	12	***13.3***
	**25700-Diarrhea**	26	***28.9***
	**26100-Dry mouth**	1	***1.1***
	**27600-Dysphagia**	1	***1.1***
	**34400-Flatulence**	1	***1.1***
	**36700-Gastrointestinal disorders - Other specify**	1	***1.1***
	**55600-Mucositis oral**	1	***1.1***
	**57600-Nausea**	19	***21.1***
	**77900-Stomach pain**	2	***2.2***
	**87900-Vomiting**	30	***33.3***

ANBL0931 Reporting Period 4: Immunotherapy ch14.18 + IL-2 + Isotretinoin

Non-Targeted Toxicities		Grade 1 or 2 (*N* = 90)	
		count	Incidence (%)
General disorders and administration site conditions	21500-Chills	6	***6.7***
	28200-Edema face	23	***25.6***
	28300-Edema limbs	11	***12.2***
	28400-Edema trunk	1	***1.1***
	33200-Fatigue	11	***12.2***
	33900-Fever	77	***85.6***
	34600-Flu like symptoms	1	***1.1***
	37200-General disorders and administration site conditions - Other specify	1	***1.1***
	45000-Infusion related reaction	2	***2.2***
	45200-Injection site reaction	1	***1.1***
	48700-Irritability	8	***8.9***
	52800-Localized edema	4	***4.4***
	54000-Malaise	1	***1.1***
	60600-Pain	44	***48.9***
Immune system disorders	12000-Allergic reaction	13	***14.4***
	24400-Cytokine release syndrome	2	***2.2***
Infections and infestations	20500-Catheter related infection	2	***2.2***
	23300-Conjunctivitis infective	1	***1.1***
	44800-Infections and infestations - Other specify	5	***5.6***
	65200-Pharyngitis	2	***2.2***
	75200-Skin infection	1	***1.1***
	82300-Upper respiratory infection	2	***2.2***
	83100-Urinary tract infection	2	***2.2***
Injury, poisoning and procedural complications	45700-Injury poisoning and procedural complications - Other specify	1	***1.1***
	66800-Postoperative hemorrhage	1	***1.1***
	86400-Vascular access complication	5	***5.6***

ANBL0931 Reporting Period 4: Immunotherapy ch14.18 + IL-2 + Isotretinoin

Non-Targeted Toxicities		Grade 1 or 2 (*N* = 90)	
		count	Incidence (%)
**Investigations**	**10900-Activated partial thromboplastin time prolonged**	2	***2.2***
	**11600-Alanine aminotransferase increased**	60	***66.7***
	**11800-Alkaline phosphatase increased**	12	***13.3***
	**15000-Aspartate aminotransferase increased**	53	***58.9***
	**17400-Blood bilirubin increased**	6	***6.7***
	**24100-Creatinine increased**	23	***25.6***
	**37500-GGT increased**	10	***11.1***
	**39400-Hemoglobin increased**	7	***7.8***
	**45800-INR increased**	1	***1.1***
	**48400-Investigations - Other specify**	1	***1.1***
	**53700-Lymphocyte count decreased**	71	***78.9***
	**53800-Lymphocyte count increased**	1	***1.1***
	**58300-Neutrophil count decreased**	45	***50.0***
	**65800-Platelet count decreased**	60	***66.7***
	**88200-Weight gain**	32	***35.6***
	**88300-Weight loss**	7	***7.8***
	**88500-White blood cell decreased**	58	***64.4***

ANBL0931 Reporting Period 4: Immunotherapy ch14.18 + IL-2 + Isotretinoin

Non-Targeted Toxicities		Grade 1 or 2 (*N* = 90)	
		count	Incidence (%)
**Metabolism and nutrition disorders**	**10700-Acidosis**	2	***2.2***
	**13500-Anorexia**	11	***12.2***
	**24700-Dehydration**	4	***4.4***
	**41300-Hypercalcemia**	6	***6.7***
	**41400-Hyperglycemia**	23	***25.6***
	**41600-Hyperkalemia**	13	***14.4***
	**41700-Hypermagnesemia**	5	***5.6***
	**41800-Hypernatremia**	1	***1.1***
	**42400-Hypertriglyceridemia**	34	***37.8***
	**42600-Hypoalbuminemia**	79	***87.8***
	**42700-Hypocalcemia**	67	***74.4***
	**42900-Hypoglycemia**	13	***14.4***
	**43100-Hypokalemia**	54	***60.0***
	**43200-Hypomagnesemia**	19	***21.1***
	**43300-Hyponatremia**	68	***75.6***
	**43500-Hypophosphatemia**	36	***40.0***
**Musculoskeletal and connective tissue disorders**	**16200-Back pain**	9	***10.0***
	**19700-Buttock pain**	1	***1.1***
	**21400-Chest wall pain**	2	***2.2***
	**37300-Generalized muscle weakness**	1	***1.1***
	**56500-Myalgia**	1	***1.1***
	**57800-Neck pain**	5	***5.6***
	**60700-Pain in extremity**	21	***23.3***
**Nervous system disorders**	**25900-Dizziness**	1	***1.1***
	**38800-Headache**	9	***10.0***
	**58200-Neuralgia**	3	***3.3***
	**64100-Peripheral sensory neuropathy**	3	***3.3***
	**76700-Somnolence**	1	***1.1***
	**81300-Tremor**	1	***1.1***
**Psychiatric disorders**	**11400-Agitation**	4	***4.4***
	**13700-Anxiety**	2	***2.2***
	**23000-Confusion**	1	***1.1***

ANBL0931 Reporting Period 4: Immunotherapy ch14.18 + IL-2 + Isotretinoin

Non-Targeted Toxicities		Grade 1 or 2 (*N* = 90) count	Grade 1 or 2 (*N* = 90) Incidence (%)
**Renal and urinary disorders**	**11100-Acute kidney injury**	1	***1.1***
	**39300-Hematuria**	14	***15.6***
	**68300-Proteinuria**	21	***23.3***
	**71000-Renal and urinary disorders - Other specify**	1	***1.1***
	**83000-Urinary retention**	9	***10.0***
	**83300-Urinary tract pain**	1	***1.1***
	**83500-Urine discoloration**	2	***2.2***
**Reproductive system and breast disorders**	**37400-Genital edema**	1	***1.1***
	**62900-Penile pain**	1	***1.1***
	**71400-Reproductive system and breast disorders - Other specify**	1	***1.1***
**Respiratory, thoracic and mediastinal disorders**	**12100-Allergic rhinitis**	2	***2.2***
	**15400-Atelectasis**	1	***1.1***
	**19300-Bronchospasm**	2	***2.2***
	**23800-Cough**	33	***36.7***
	**27800-Dyspnea**	2	***2.2***
	**29700-Epistaxis**	3	***3.3***
	**40700-Hiccups**	1	***1.1***
	**43900-Hypoxia**	12	***13.3***
	**51500-Laryngopharyngeal dysesthesia**	1	***1.1***
	**57500-Nasal congestion**	9	***10.0***
	**65900-Pleural effusion**	2	***2.2***
	**71600-Respiratory thoracic and mediastinal disorders - Other specify**	15	***16.7***
	**76800-Sore throat**	2	***2.2***
	**88400-Wheezing**	6	***6.7***

ANBL0931 Reporting Period 4: Immunotherapy ch14.18 + IL-2 + Isotretinoin

Non-Targeted Toxicities		Grade 1 or 2 (*N* = 90)	
		count	Incidence (%)
**Skin and subcutaneous tissue disorders**	**12200-Alopecia**	2	***2.2***
	**26200-Dry skin**	21	***23.3***
	**29900-Erythema multiforme**	1	***1.1***
	**63600-Periorbital edema**	8	***8.9***
	**68400-Pruritus**	33	***36.7***
	**69700-Rash maculo-papular**	23	***25.6***
	**74700-Skin and subcutaneous tissue disorders - Other specify**	11	***12.2***
	**84100-Urticaria**	19	***21.1***
**Vascular disorders**	**19800-Capillary leak syndrome**	29	***32.2***
	**34700-Flushing**	2	***2.2***
	**42100-Hypertension**	29	***32.2***
	**43600-Hypotension**	56	***62.2***
	**86500-Vascular disorders - Other specify**	2	***2.2***

ANBL0931 Reporting Period 5: Immunotherapy ch14.18 + GM-CSF + Isotretinoin

Non-Targeted Toxicities		Grade 1 or 2 (*N* = 88)	
		count	Incidence (%)
**Primary Category**			
**Blood and lymphatic system disorders**	**13200-Anemia**	64	***72.7***
**Cardiac disorders**	**20100-Cardiac disorders - Other specify**	1	***1.1***
	**74200-Sinus bradycardia**	1	***1.1***
	**74500-Sinus tachycardia**	51	***58.0***
	**78900-Supraventricular tachycardia**	1	***1.1***
**Ear and labyrinth disorders**	**28000-Ear pain**	1	***1.1***
**Endocrine disorders**	**43800-Hypothyroidism**	1	***1.1***
**Eye disorders**	**31900-Eye disorders - Other specify**	1	***1.1***
	**61900-Papilledema**	1	***1.1***
	**65600-Photophobia**	1	***1.1***
**Gastrointestinal disorders**	**10100-Abdominal distension**	3	***3.4***
	**10300-Abdominal pain**	25	***28.4***
	**23400-Constipation**	7	***8.0***
	**25700-Diarrhea**	14	***15.9***
	**27500-Dyspepsia**	1	***1.1***
	**27600-Dysphagia**	1	***1.1***
	**34400-Flatulence**	2	***2.3***
	**36400-Gastritis**	1	***1.1***
	**36700-Gastrointestinal disorders - Other specify**	1	***1.1***
	**55600-Mucositis oral**	1	***1.1***
	**57600-Nausea**	16	***18.2***
	**77900-Stomach pain**	1	***1.1***
	**80200-Toothache**	1	***1.1***
	**87900-Vomiting**	24	***27.3***

ANBL0931 Reporting Period 5: Immunotherapy ch14.18 + GM-CSF + Isotretinoin

Non-Targeted Toxicities		Grade 1 or 2 (*N* = 88)	
		count	Incidence (%)
General disorders and administration site conditions	28200-Edema face	7	***8.0***
	28300-Edema limbs	7	***8.0***
	33200-Fatigue	8	***9.1***
	33900-Fever	59	***67.0***
	45200-Injection site reaction	10	***11.4***
	48700-Irritability	8	***9.1***
	52800-Localized edema	1	***1.1***
	60600-Pain	39	***44.3***
Immune system disorders	12000-Allergic reaction	9	***10.2***
Infections and infestations	20500-Catheter related infection	1	***1.1***
	29500-Enterocolitis infectious	2	***2.3***
	44800-Infections and infestations - Other specify	2	***2.3***
	55500-Mucosal infection	1	***1.1***
	60100-Otitis media	1	***1.1***
	65200-Pharyngitis	1	***1.1***
	72600-Rhinitis infective	1	***1.1***
	75200-Skin infection	2	***2.3***
	82300-Upper respiratory infection	1	***1.1***
	83100-Urinary tract infection	2	***2.3***
Injury, poisoning and procedural complications	19400-Bruising	1	***1.1***

ANBL0931 Reporting Period 5: Immunotherapy ch14.18 + GM-CSF + Isotretinoin

Non-Targeted Toxicities		Grade 1 or 2 (*N* = 88)	
		count	Incidence (%)
**Investigations**	**10900-Activated partial thromboplastin time prolonged**	1	***1.1***
	**11600-Alanine aminotransferase increased**	32	***36.4***
	**11800-Alkaline phosphatase increased**	5	***5.7***
	**15000-Aspartate aminotransferase increased**	15	***17.0***
	**17400-Blood bilirubin increased**	2	***2.3***
	**24100-Creatinine increased**	15	***17.0***
	**37500-GGT increased**	6	***6.8***
	**39400-Hemoglobin increased**	5	***5.7***
	**45800-INR increased**	1	***1.1***
	**53700-Lymphocyte count decreased**	41	***46.6***
	**53800-Lymphocyte count increased**	1	***1.1***
	**58300-Neutrophil count decreased**	15	***17.0***
	**65800-Platelet count decreased**	44	***50.0***
	**88200-Weight gain**	29	***33.0***
	**88300-Weight loss**	3	***3.4***
	**88500-White blood cell decreased**	21	***23.9***

ANBL0931 Reporting Period 5: Immunotherapy ch14.18 + GM-CSF + Isotretinoin

Non-Targeted Toxicities		Grade 1 or 2 (*N* = 88)	
		count	Incidence (%)
**Metabolism and nutrition disorders**	**10700-Acidosis**	1	***1.1***
	**13500-Anorexia**	7	***8.0***
	**24700-Dehydration**	3	***3.4***
	**41300-Hypercalcemia**	3	***3.4***
	**41400-Hyperglycemia**	17	***19.3***
	**41600-Hyperkalemia**	5	***5.7***
	**41700-Hypermagnesemia**	6	***6.8***
	**41800-Hypernatremia**	3	***3.4***
	**42400-Hypertriglyceridemia**	19	***21.6***
	**42500-Hyperuricemia**	2	***2.3***
	**42600-Hypoalbuminemia**	65	***73.9***
	**42700-Hypocalcemia**	37	***42.0***
	**42900-Hypoglycemia**	11	***12.5***
	**43100-Hypokalemia**	26	***29.5***
	**43200-Hypomagnesemia**	11	***12.5***
	**43300-Hyponatremia**	46	***52.3***
	**43500-Hypophosphatemia**	9	***10.2***
**Musculoskeletal and connective tissue disorders**	**16200-Back pain**	9	***10.2***
	**18200-Bone pain**	1	***1.1***
	**21400-Chest wall pain**	2	***2.3***
	**56300-Musculoskeletal and connective tissue disorder - Other specify**	1	***1.1***
	**57800-Neck pain**	4	***4.5***
	**60700-Pain in extremity**	9	***10.2***
**Nervous system disorders**	**38800-Headache**	7	***8.0***
	**51800-Lethargy**	2	***2.3***
	**54500-Meningismus**	1	***1.1***
	**55400-Movements involuntary**	2	***2.3***
	**58200-Neuralgia**	1	***1.1***
	**58700-Nystagmus**	1	***1.1***
	**59000-Oculomotor nerve disorder**	1	***1.1***
	**81300-Tremor**	1	***1.1***

ANBL0931 Reporting Period 5: Immunotherapy ch14.18 + GM-CSF + Isotretinoin

Non-Targeted Toxicities		Grade 1 or 2 (*N* = 88) count	Grade 1 or 2 (*N* = 88) Incidence (%)
**Psychiatric disorders**	**11400-Agitation**	4	***4.5***
	**13700-Anxiety**	1	***1.1***
	**23000-Confusion**	1	***1.1***
	**25400-Depression**	1	***1.1***
	**38500-Hallucinations**	1	***1.1***
	**45900-Insomnia**	1	***1.1***
	**71700-Restlessness**	1	***1.1***
**Renal and urinary disorders**	**39300-Hematuria**	8	***9.1***
	**68300-Proteinuria**	12	***13.6***
	**71000-Renal and urinary disorders - Other specify**	1	***1.1***
	**83000-Urinary retention**	6	***6.8***
	**83300-Urinary tract pain**	1	***1.1***
	**83500-Urine discoloration**	1	***1.1***
**Reproductive system and breast disorders**	**37400-Genital edema**	1	***1.1***
	**71400-Reproductive system and breast disorders - Other specify**	1	***1.1***
**Respiratory, thoracic and mediastinal disorders**	**12100-Allergic rhinitis**	1	***1.1***
	**23800-Cough**	21	***23.9***
	**27800-Dyspnea**	1	***1.1***
	**43900-Hypoxia**	14	***15.9***
	**57500-Nasal congestion**	3	***3.4***
	**66300-Pneumonitis**	2	***2.3***
	**67600-Productive cough**	1	***1.1***
	**71600-Respiratory thoracic and mediastinal disorders - Other specify**	11	***12.5***
	**76200-Sneezing**	2	***2.3***
	**88400-Wheezing**	2	***2.3***

ANBL0931 Reporting Period 5: Immunotherapy ch14.18 + GM-CSF + Isotretinoin

Non-Targeted Toxicities		Grade 1 or 2 (*N* = 88)	
		count	Incidence (%)
**Skin and subcutaneous tissue disorders**	**26200-Dry skin**	11	***12.5***
	**60900-Palmar-plantar erythrodysesthesia syndrome**	1	***1.1***
	**63600-Periorbital edema**	4	***4.5***
	**65700-Photosensitivity**	1	***1.1***
	**68400-Pruritus**	28	***31.8***
	**69600-Rash acneiform**	1	***1.1***
	**69700-Rash maculo-papular**	13	***14.8***
	**74700-Skin and subcutaneous tissue disorders - Other specify**	2	***2.3***
	**75100-Skin induration**	2	***2.3***
	**75300-Skin ulceration**	1	***1.1***
	**84100-Urticaria**	8	***9.1***
**Vascular disorders**	**19800-Capillary leak syndrome**	15	***17.0***
	**42100-Hypertension**	25	***28.4***
	**43600-Hypotension**	42	***47.7***
	**86500-Vascular disorders - Other specify**	1	***1.1***

ANBL0931 Reporting Period 6: Isotretinoin (ISOT) only

Non-Targeted Toxicities		Grade 1 or 2 (*N* = 81)	
		count	Incidence (%)
**Primary Category**			
**Blood and lymphatic system disorders**	**13200-Anemia**	39	***48.1***
**Cardiac disorders**	**74500-Sinus tachycardia**	9	***11.1***
**Ear and labyrinth disorders**	**28000-Ear pain**	1	***1.2***
**Endocrine disorders**	**42200-Hyperthyroidism**	3	***3.7***
**Eye disorders**	**23200-Conjunctivitis**	1	***1.2***
	**26000-Dry eye**	2	***2.5***
	**31900-Eye disorders - Other specify**	1	***1.2***
	**32200-Eyelid function disorder**	1	***1.2***
**Gastrointestinal disorders**	**10300-Abdominal pain**	5	***6.2***
	**23400-Constipation**	1	***1.2***
	**25700-Diarrhea**	5	***6.2***
	**57600-Nausea**	2	***2.5***
	**77900-Stomach pain**	1	***1.2***
	**87900-Vomiting**	4	***4.9***
**General disorders and administration site conditions**	**21500-Chills**	2	***2.5***
	**33200-Fatigue**	7	***8.6***
	**33900-Fever**	15	***18.5***
	**35000-Gait disturbance**	1	***1.2***
	**48700-Irritability**	2	***2.5***
	**58600-Non-cardiac chest pain**	2	***2.5***
	**60600-Pain**	5	***6.2***
**Infections and infestations**	**29500-Enterocolitis infectious**	1	***1.2***
	**44800-Infections and infestations - Other specify**	7	***8.6***
	**60100-Otitis media**	3	***3.7***
	**62000-Papulopustular rash**	1	***1.2***
	**74600-Sinusitis**	1	***1.2***
	**75200-Skin infection**	1	***1.2***
	**82300-Upper respiratory infection**	1	***1.2***
**Injury, poisoning and procedural complications**	**19400-Bruising**	1	***1.2***
	**86400-Vascular access complication**	1	***1.2***

ANBL0931 Reporting Period 6: Isotretinoin (ISOT) only

Non-Targeted Toxicities		Grade 1 or 2 (*N* = 81)	
		count	Incidence (%)
**Investigations**	**10900-Activated partial thromboplastin time prolonged**	2	***2.5***
	**11600-Alanine aminotransferase increased**	24	***29.6***
	**11800-Alkaline phosphatase increased**	3	***3.7***
	**15000-Aspartate aminotransferase increased**	23	***28.4***
	**24100-Creatinine increased**	11	***13.6***
	**37500-GGT increased**	5	***6.2***
	**45800-INR increased**	1	***1.2***
	**53700-Lymphocyte count decreased**	16	***19.8***
	**58300-Neutrophil count decreased**	24	***29.6***
	**65800-Platelet count decreased**	18	***22.2***
	**88200-Weight gain**	4	***4.9***
	**88300-Weight loss**	2	***2.5***
	**88500-White blood cell decreased**	35	***43.2***
**Metabolism and nutrition disorders**	**10700-Acidosis**	1	***1.2***
	**13500-Anorexia**	6	***7.4***
	**24700-Dehydration**	1	***1.2***
	**41300-Hypercalcemia**	4	***4.9***
	**41400-Hyperglycemia**	5	***6.2***
	**41600-Hyperkalemia**	8	***9.9***
	**41700-Hypermagnesemia**	1	***1.2***
	**41800-Hypernatremia**	2	***2.5***
	**42400-Hypertriglyceridemia**	15	***18.5***
	**42600-Hypoalbuminemia**	3	***3.7***
	**42700-Hypocalcemia**	2	***2.5***
	**42900-Hypoglycemia**	5	***6.2***
	**43100-Hypokalemia**	1	***1.2***
	**43200-Hypomagnesemia**	3	***3.7***
	**43300-Hyponatremia**	10	***12.3***
	**43500-Hypophosphatemia**	2	***2.5***
**Musculoskeletal and connective tissue disorders**	**18200-Bone pain**	1	***1.2***
	**56500-Myalgia**	2	***2.5***
	**60700-Pain in extremity**	3	***3.7***

ANBL0931 Reporting Period 6: Isotretinoin (ISOT) only

Non-Targeted Toxicities		Grade 1 or 2 (*N* = 81) count	Incidence (%)
**Nervous system disorders**	**38800-Headache**	4	***4.9***
	**51800-Lethargy**	1	***1.2***
	**64100-Peripheral sensory neuropathy**	1	***1.2***
**Psychiatric disorders**	**13700-Anxiety**	1	***1.2***
**Renal and urinary disorders**	**39300-Hematuria**	5	***6.2***
	**68300-Proteinuria**	7	***8.6***
	**71000-Renal and urinary disorders - Other specify**	1	***1.2***
**Reproductive system and breast disorders**	**85600-Vaginal inflammation**	1	***1.2***
**Respiratory, thoracic and mediastinal disorders**	**12100-Allergic rhinitis**	3	***3.7***
	**15400-Atelectasis**	2	***2.5***
	**23800-Cough**	9	***11.1***
	**29700-Epistaxis**	1	***1.2***
	**43900-Hypoxia**	1	***1.2***
	**51600-Laryngospasm**	1	***1.2***
	**57500-Nasal congestion**	5	***6.2***
	**65300-Pharyngolaryngeal pain**	1	***1.2***
	**67600-Productive cough**	1	***1.2***
	**71600-Respiratory thoracic and mediastinal disorders - Other specify**	7	***8.6***
	**76800-Sore throat**	5	***6.2***
	**88400-Wheezing**	1	***1.2***
**Skin and subcutaneous tissue disorders**	**26200-Dry skin**	18	***22.2***
	**29900-Erythema multiforme**	1	***1.2***
	**68400-Pruritus**	4	***4.9***
	**69600-Rash acneiform**	1	***1.2***
	**69700-Rash maculo-papular**	3	***3.7***
	**74700-Skin and subcutaneous tissue disorders - Other specify**	1	***1.2***
**Vascular disorders**	**42100-Hypertension**	4	***4.9***
	**43600-Hypotension**	11	***13.6***

Unacceptable toxicity grading according to the Common Terminology Criteria for Adverse Events (version 4.0):

Neuropathic pain codes: 58200-Neuralgia, 60700-Pain in extremity, 16200-Back pain ,60600-Pain, 10300-Abdominal pain
Anatomical pain codes: listed in separate attachment

19800-Capillary leak syndrome,

12000-Allergic reaction, 13100- Anaphylaxis,

42100-hypertension,

33900-Fever

Ocular tox codes: 65600-Photophobia, 31900-Eye disorders - Other, specify, 17800-Blurred vision
