# Supplementary material for: A Comprehensive Safety Trial of Chimeric Antibody 14.18 With GM-CSF, IL-2, and Isotretinoin in High-Risk Neuroblastoma Patients Following Myeloablative Therapy: Children’s Oncology Group Study ANBL0931
Source: Front Immunol. 2018 Jun 18;9:1355. doi: 10.3389/fimmu.2018.01355 (PMC6016521; doi:10.3389/fimmu.2018.01355)
Supplement: Supplementary file 2 [file table_2.PDF]

**Supplemental Table 2 (Accompanying Fig. 1A and 1B). Cytokine, chemokine and nitrate levels in ANBL0931 patients and normal adults.**

| | **IL-6** | **[f]IL-1ra** | **[g]CCL22** | **IFNɣ** | **IL-10** | **TNFα** | **IL-4** | **IL-5** | **IL12p70** | **IL-17A** | **CXCL9** | **IL-8** | **IL-15** | **IL-18** | **nitrate** |
|---|---|---|---|---|---|---|---|---|---|---|---|---|---|---|---|
| **[a]C1, day -1<br>(pretreatment<br>Range)<br><br>Sample size** | 14.1 ±<br>65.0<br>0.5 -<br>447.4<br>N=87 | 905.2<br>± 117.7<br>258.0-<br>9605.8<br>N=89 | 2201.6<br>± 125.1<br>788.7-<br>>3200<br>N=87 | 4.9<br>± 0.6<br>0.5-<br>29.8<br>N=87 | 2.7<br>± 0.5<br>0.4-<br>44.3<br>N=87 | 11.6<br>± 3.1<br>2.0-<br>268.5<br>N=87 | 0.14<br>± 0.01<br>0.03-<br>0.78<br>N=83 | 1.16<br>± 0.14<br>0.08-<br>8.0<br>N=83 | 0.97<br>± 0.08<br>0.0-<br>5.5<br>N=83 | 1.32<br>± 0.28<br>0.05-<br>22.4<br>N=83 | 492.8<br>± 55.2<br>6.3-<br>3184.7<br>N=80 | [h]42.3<br>± 14.0<br>0.4-<br>878<br>N=79 | 2.3<br>± 0.1<br>0.0-<br>7.8<br>N=80 | 534.4<br>± 36.6<br>12.0-<br>1667.5<br>N=80 | 29.7<br>± 1.9<br>2.2-<br>130.4<br>N=98 |
| **C1, day 6<br>(all pts)<br>Range<br><br>Sample size<br>[b]p=d-1 vs d6** | 30.5<br>± 8.8<br>3.4 –<br>618.6<br>N=70<br>**p<0.001** | 2166.8<br>± 180.8<br>522 –<br>11489<br>N=74<br>**p<0.001** | 6070.6<br>± 633.4<br>893 –<br>39957<br>N=66<br>[i]na | 45.9<br>± 7.5<br>1.2 –<br>317<br>N=68<br>**p<0.001** | 4.3<br>± 0.5<br>0.8 –<br>21.1<br>N=68<br>**p<0.001** | 11.5<br>± 1.4<br>2.5 –<br>70.6<br>N=68<br>**p<0.001** | 0.14<br>± 0.01<br>0.01 –<br>0.3<br>N=45<br>p>0.05 | 965.3<br>± 153.2<br>12.3 –<br>5115<br>N=46<br>**p<0.001** | 0.67<br>± 0.05<br>0.04 –<br>1.7<br>N=46<br>p>0.05 | 1.98<br>± 0.30<br>0.28 –<br>8.3<br>N=46<br>**p<0.001** | 868.4<br>± 118.4<br>133 -<br>3896<br>N=41<br>**p<0.001** | 89.4<br>± 39.2<br>8.9 -<br>1385<br>N=41<br>p>0.05 | 4.62<br>± 0.45<br>0.6 –<br>19.2<br>N=41<br>**p<0.001** | 452.1<br>± 30.7<br>133 -<br>1072<br>N=41<br>p>0.05 | 23.6<br>± 1.8<br>2.8 –<br>106.4<br>N=88<br>**p<0.01** |
| **C1, day 6<br>(No [c]AR)<br>Sample size** | 21.9<br>± 2.83<br>N=50 | 2043.9<br>± 226.9<br>N=55 | 6222.3<br>± 832.1<br>N=49 | 35.9<br>± 7.1<br>N=50 | 3.95<br>± 0.46<br>N=50 | 9.61<br>± 0.65<br>N=50 | 0.14<br>± 0.01<br>N=33 | 1011.3<br>± 200.1<br>N=34 | 0.69<br>± 0.06<br>N=34 | 1.80<br>± 0.32<br>N=34 | 877.5<br>± 148.7<br>N=30 | 45.5<br>± 19.7<br>N=30 | 4.80<br>± 0.59<br>N=30 | 466.0<br>± 37.8<br>N=30 | na |
| **C1, day 6<br>(AR)<br>Sample size<br>[d]p=no AR vs AR** | 52.1<br>± 30.0<br>N=20<br>p>0.05 | 2522.3<br>± 245.9<br>N=19<br>**^p<0.05** | 5633.3<br>± 570.6<br>N=17<br>p>0.05 | 73.8<br>± 19.1<br>N=18<br>**^p<0.05** | 5.2<br>± 1.2<br>N=18<br>p>0.05 | 16.6<br>± 4.7<br>N=18<br>p>0.05 | 0.14<br>± 0.01<br>N=12<br>p>0.05 | 834.8<br>± 160.06<br>N=12<br>p>0.05 | 0.60<br>± 0.12<br>N=12<br>p>0.05 | 2.49<br>± 0.74<br>N=12<br>p>0.05 | 843.4<br>± 184.4<br>N=11<br>p>0.05 | 208.9<br>± 133.9<br>N=11<br>p>0.05 | 4.15<br>± 0.37<br>N=11<br>p>0.05 | 414.3<br>± 50.8<br>N=11<br>p>0.05 | na |
| **C4, day 80<br>(all pts)<br>Range<br><br>Sample size** | 16.3<br>±12.8<br>0.35-<br>861.99<br>N=67 | 838.3<br>± 56.7<br>255.1-<br>2540.2<br>N=70 | 3409.3<br>± 217.2<br>697.9-<br>11504<br>N=64 | 4.18<br>± 1.29<br>0.17-<br>77.1<br>N=64 | 3.54<br>± 0.79<br>0.57-<br>50.2<br>N=64 | 8.16<br>± 0.50<br>1.80-<br>27.2<br>N=64 | 0.13<br>± 0.01<br>0.01-<br>0.30<br>N=46 | 50.6<br>± 36.0<br>0.00-<br>1452.1<br>N=46 | 0.63<br>± 0.04<br>0.07-<br>1.2<br>N=45 | 0.95<br>± 0.11<br>0.00-<br>3.1<br>N=45 | 424.7<br>± 63.71<br>116.46-<br>2911.5<br>N=50 | 73.9<br>± 43.78<br>2.89-<br>2182.5<br>N=50 | 2.5<br>± 0.72<br>0.04-<br>33.7<br>N=50 | 431.3<br>± 27.95<br>154.19-<br>1106.0<br>N=50 | 29.7<br>± 1.6<br>4.0-<br>>72.7<br>N=81 |
| **C4, day 90<br>(all pts)<br>Range<br><br>Sample size<br>[b]p=d80 vs d90** | 36.8<br>± 4.5<br>0.93 –<br>187<br>N=63<br>**p<0.001** | 2659.2<br>± 256.7<br>410 –<br>13330<br>N=70<br>**p<0.001** | 5547.2<br>± 418.7<br>1701 –<br>17238<br>N=61<br>na | 474.7<br>± 169.1<br>0.61 –<br>9592<br>N=62<br>**p<0.001** | 34.9<br>± 3.9<br>2.5 –<br>194<br>N=62<br>**p<0.001** | 18.8<br>± 1.2<br>5.7 –<br>54.4<br>N=62<br>**p<0.001** | 0.17<br>± 0.01<br>0.05 –<br>0.47<br>N=46<br>**p<0.001** | 1304.9<br>± 228.7<br>3.6 –<br>7501<br>N=46<br>**p<0.001** | 0.59<br>± 0.05<br>0.01 –<br>1.74<br>N=46<br>p>0.05 | 2.19<br>± 0.47<br>0.17 –<br>20.7<br>N=46<br>**p<0.001** | 3999.5<br>± 648.3<br>586 –<br>24173<br>N=49<br>**p<0.001** | 66.4<br>± 24.0<br>5.0 –<br>1169<br>N=49<br>p>0.05 | 4.04<br>± 0.69<br>0.09 –<br>33.4<br>N=49<br>**p<0.001** | 680.1<br>± 51.0<br>87.6 –<br>1763<br>N=49<br>**p<0.001** | 28.1<br>± 2.3<br>5.9 –<br>120.3<br>N=77<br>p>0.05 |
| **C4, day 90<br>(No AR)<br>Sample size** | 39.3<br>± 6.0<br>N=41 | 2591.8<br>± 314.9<br>N=46 | 5654.7<br>± 547.1<br>N=40 | 534.6<br>± 251.5<br>N=40 | 34.6<br>± 3.3<br>N=40 | 19.9<br>± 1.5<br>N=40 | 0.17<br>± 0.01<br>N=32 | 1336.1<br>± 292.6<br>N=32 | 0.62<br>± 0.06<br>N=32 | 1.77<br>± 0.27<br>N=32 | 3404.1<br>± 473.0<br>N=33 | 78.2<br>± 34.8<br>N=33 | 4.16<br>± 0.96<br>N=33 | 704.3<br>± 54.0<br>N=33 | na |

| | | | | | | | | | | | | | | | |
|---|---|---|---|---|---|---|---|---|---|---|---|---|---|---|---|
| **C4, day 90 (AR) Sample size p=no AR vs AR** | 32.1 ± 6.3 N=22 p>0.50 | 2788.2 ± 451.2 N=24 p>0.50 | 5342.2 ± 642.7 N=21 p>0.50 | 365.9 ± 139.8 N=22 p>0.50 | 35.4 ± 9.3 N=22 p>0.50 | 17.0 ± 2.0 N=22 p>0.50 | 0.16 ± 0.02 N=14 p>0.50 | 1233.6 ± 357.2 N=14 p>0.50 | 0.50 ± 0.06 N=14 p>0.50 | 3.16 ± 1.42 N=14 p>0.50 | 5227.5 ± 1727.9 N=16 p>0.50 | 42.2 ± 16.7 N=16 p>0.50 | 3.80 ± 0.79 N=16 p>0.50 | 630.3 ± 111.5 N=16 p>0.50 | na |
| [e]**Normal adults Range Sample size** | 0.68 ± 0.11 0.2 – 1.0 N=6 | 667.5 ±39.1 510.4 – 753.2 N=6 | 1393.8 ± 76 1318 – 1470 N=2 | 0.71 ± 0.06 0.65 – 0.78 N=2 | 1.5 ± 1.1 0.40 – 2.59 N=2 | 5.3 ± 2.7 2.59 – 7.96 N=2 | 0.09 ± 0.02 0.06 – 0.11 N=3 | 0.36 ± 0.11 0.17 – 0.55 N=3 | 0.30 ± 0.16 0.03 – 0.57 N=3 | 0.04 ±0.04 0.0 – 0.12 N=3 | 156.6 ± 42.4 112.5 – 241.3 N=3 | 13.5 ± 4.2 7.3 – 21.4 N=3 | 1.3 ± 0.23 0.84 – 1.60 N=3 | 361.9 ±51.2 260.2 – 422.4 N=-3 | [k]24.8 ± 2.73 N=19 |
| **C4, day 90 anaphylaxis pt (N=1) mean ± SD** | 25.1 ± [j]nd | 1278.6 ± 13.7 | 10530 ± 195.1 | 32.9 ±0.3 | 24.7 ± 0.1 | 13.2 ± 0.5 | 0.16 ± 0.01 | 1635.0 ± 59.0 | 0.45 ± 0.13d | 4.02 ± 0.14 | 3458.7 ± 53.3 | 16.8 ± 0.06 | 4.24 ± 0.06 | 651.4 ± 8.5 | na |

[a]Course 1, day 6 (C1, d6) and Course 4, day 90 (C4, d90). Cytokine values in pg/ml, nitrate in uM. Data are presented as mean ± standard error except where noted.

[b]Wilcoxon signed-rank test comparison of paired samples: day -1 versus day 6 and day 80 versus day 90. A p-value of <0.0036 is considered statistically significant at level 0.05 and are Bonferroni corrected for the 14 cytokine comparisons at each course.

[c]AR: Allergic Reaction toxicities are anaphylaxsis, urticaria, wheezing, stridor, bronchospasm and generally diagnosed allergic reaction;

[d] Two-sided Mann-Whitnet U-test comp arison of no AR versus any AR, values are not adjusted for multiple comparisons.

[e]Normal cytokine and chemokine levels were determined from healthy adult volunteers.

[f] Interleukin-1 receptor antagonist (IL-1Ra)

[g]CCL22 data were out of range of the multiplex assay and were extrapolated and thus not statistically analyzed.

[h]IL-8 was significantly above the median in a single patient. This day -1 outlier (31,818 pg/ml) was excluded from the calculation of the mean and standard error.

[i]na; not applicable (due to a lack of scientific rationale);

[j]nd; not determined (due to lack of sufficient sample);

[k]Data from reference #15.

^IFN p = 0.023, IL1Ra, p=0.017
