# Supplementary material for: A Comprehensive Safety Trial of Chimeric Antibody 14.18 With GM-CSF, IL-2, and Isotretinoin in High-Risk Neuroblastoma Patients Following Myeloablative Therapy: Children’s Oncology Group Study ANBL0931
Source: Front Immunol. 2018 Jun 18;9:1355. doi: 10.3389/fimmu.2018.01355 (PMC6016521; doi:10.3389/fimmu.2018.01355)
Supplement: Supplementary file 3 [file data_sheet_1.doc]

**APPENDIX**

**NAME OF THE PARTICIPATING INSTITUTIONS**

1 Children’s of New Orleans/LSUMC CCOP

2 Children’s Hospital of Orange County

3 Children’s Hospital of Philadelphia

4 Cincinnati Children’s Hospital Medical Center

5 Cook Children’s Medical Center

6 Dana-Farber Cancer Institute and Children’s Hospital

7 New York Medical College

8 Rady Children’s Hospital San Diego

9 Seattle Children’s

10 St. Jude Children’s Research Hospital Memphis

11 Texas Children’s Cancer Center at Baylor College of Medicine

12 The University of Chicago Comer Children’s Hospital

13 University of Wisconsin - AFCH

14 Vanderbilt Children’s Hospital

15 Washington University Medical Center

16 C.S Mott Children’s Hospital

17 Indiana University – Riley Children’s Hospital

18 The Children’s Mercy Hospital

19 UT Southwestern Medical Center

20 Stanford University Medical Center

21 Children’s Hospital Los Angeles

22 University of Minnesota Cancer Center

23 The Children’s Hospital, Denver, Colorado

24 UCSF School of Medicine

25 Loma Linda University Medical Center

26 Children’s Hospitals and Clinics of Minnesota

27 Children’s Healthcare of Atlanta, Emory University

28 City of Hope National Medical Center

29 Duke University Medical Center

30 Columbia Presbyterian College of Physicians & Surgeons

INCLUSION CRITERIA

3.2.1 Age

There are no restrictions on the age of the patient at diagnosis.

3.2.2 Diagnosis

3.2.2.1

All patients must be diagnosed with neuroblastoma, and categorized as high-risk at the time of diagnosis.

3.2.2.2

At pre-ASCT evaluation, patients must meet the International Neuroblastoma Response Criteria (INRC) for CR, VGPR, or PR for primary site, soft tissue metastases and bone metastases. Patients who meet those criteria must also meet the protocol specified criteria for bone marrow response as outlined below: ≤ 10% tumor (of total nucleated cellular content) seen on any specimen from a bilateral bone marrow aspirate/biopsy. Patients who have no tumor seen on the prior bone marrow, and then have ≤ 10% tumor on any of the bilateral marrow aspirate/biopsy specimens done at pre-ASCT and/or pre-enrollment evaluation will also be eligible. (Note that, per INRC, this would have been defined as an “overall” response of PD).

3.2.2.3

Pre-enrollment tumor survey:

Prior to enrollment on ANBL0931, a determination of residual disease must be performed (tumor imaging studies including MIBG scan, CT or MRI, bone marrow aspiration and biopsy). **This disease assessment is required for eligibility**, and should be done preferably within 2 weeks but must be done within a maximum of 4 weeks before enrollment. (**See Section 14.0 for further details of imaging requirements)**. Patients with residual disease are eligible. Biopsy is not required.

Patients must not have progressive disease except for protocol specified bone marrow response as

defined in Section 3.2.2.2.

3.2.3 Prior Therapy

3.2.3.1

All patients must have completed therapy including intensive induction chemotherapy followed by ASCT and radiotherapy to be eligible. Radiotherapy may be waived for patients who either had a small adrenal mass which was completely resected upfront, or who never had an identifiable primary tumor.

3.2.3.2

No more than 9 months from the date of starting the first induction chemotherapy after diagnosis to the date of ASCT except for the rare occasions as noted below. For tandem ASCT patients, this will be the date of the FIRST stem cell infusion. **Exception: For those who are initially diagnosed as non-high risk neuroblastoma, but later converted (and/ or relapsed) to high risk neuroblastoma, the 9 months restriction should start from the date of induction therapy for high risk neuroblastoma (not from the initial induction therapy for non-high risk disease), to the date of ASCT.**

3.2.4 Performance Level

(See https://members.childrensoncologygroup.org/prot/reference_materials.asp

under Standard Material For Protocols). Use Karnofsky for patients >16 years of age and Lansky for patients ≤ 16 years of age. Required Patients must have a Lansky or Karnofsky performance scale score of ≥ 50%.

3.2.5 Life Expectancy

Patients must have a life expectancy of ≥ 2 months (8 weeks).

3.2.6 Organ Function Requirements:

All patients must have adequate organ function as described below:

3.2.6.1 Hematological:

Total absolute phagocyte count (APC = neutrophils + monocytes) is at least 1000/ L.

3.2.6.2 Adequate Renal Function Defined As:

- Creatinine clearance or radioisotope GFR 70mL/min/1.73 m2 or

- A serum creatinine based on age/gender as follows:

**Age Maximum Serum**

**Creatinine (mg/dL)**

**Male Female**

1 month to < 6 months 0.4 0.4

6 months to < 1 year 0.5 0.5

1 to < 2 years 0.6 0.6

2 to < 6 years 0.8 0.8

6 to < 10 years 1 1

10 to < 13 years 1.2 1.2

13 to < 16 years 1.5 1.4

≥ 16 years 1.7 1.4

(The threshold creatinine values in this Table were derived from the Schwartz formula for

estimating GFR (Schwartz et al. J. Peds, 106:522, 1985) utilizing child length and stature

data published by the CDC)

3.2.6.3 Adequate liver function defined as:

- Total bilirubin ≤ 1.5 x upper limit of normal (ULN) for age, and

- SGPT (ALT) < 5 x upper limit of normal (ULN) for age.

- SOS (sinusoidal obstruction syndrome, formerly known as veno-occlusive disease [VOD]), if

present, should be stable or improving.

3.2.6.4 Adequate cardiac function defined as:

- Shortening fraction of 27% by echocardiogram, or

- Ejection fraction of 55% by radionuclide angiography.

3.2.6.5 Adequate pulmonary function defined as:

- No evidence of dyspnea at rest.

- If PFTs are performed, FEV1/FVC > 60% by pulmonary function test.

3.2.6.6 Central Nervous System Function Defined As:

- Patients with seizure disorder may be enrolled if on anticonvulsants and well controlled.

- CNS toxicity < Grade 2.

EXCLUSION CRITERIA

3.2.7 Females of childbearing potential must have a negative pregnancy test.

3.2.8 Patients of childbearing potential must agree to use an effective birth control method.

3.2.9 Female patients who are lactating must agree to stop breast-feeding.

3.2.10 Patients must not have received prior anti-GD2 antibody therapy.

3.2.11 Patients must not have received prior vaccine therapy administered as treatment of

neuroblastoma **not** routine infectious disease vaccinations.

**Supportive Care**

**Please note the maximum infusion time from initiation of ch14.18 is 20 hours even if the total dose has not been administered in that timeframe.**

Premedication and Supportive Care for the prevention of anticipated toxicities associated with ch14.18:

Neuropathic pain and allergic reactions with angioedema are commonly seen in patients receiving

monoclonal antibody. The following premedications will be utilized to avoid these problems:

a. Hydroxyzine (0.5-1 mg/kg; max dose 50 mg) OR diphenhydramine (0.5-1 mg/kg; max. dose

50 mg) IV over 10-15 minutes to start 20 minutes prior to ch14.18 infusion and continue these

agents, as tolerated every 4-6 hours until end of ch14.18 infusion (doses may be skipped at

discretion of treating physician if child is over sedated)

b. Acetaminophen (10 mg/kg; max. dose 650 mg) PO given 20 minutes prior to ch14.18 infusion;

may be given every 4 hours prn fever. Administer acetaminophen scheduled every 4-6 hours

during IL-2 plus ch14.18 courses. May administer ibuprofen (5-10 mg/kg/dose, not to exceed

every 6 hours) between acetaminophen doses for control of persistent fever provided that there is

no ongoing bleeding problem and the platelet count is >50,000/μL, and no renal dysfunction. If

renal dysfunction occurs or bleeding occurs, stop ibuprofen.

c. Meperidine 0.25- 0.5mg/kg IV (max. dose 50 mg) every 2 hours PRN chills.

d. Give a morphine sulfate loading dose of 50 micrograms/kg immediately prior to ch14.18

administration and then continue with morphine sulfate drip with an infusion rate of

20-50 micrograms/kg/hour to continue for two hours after completion of the ch14.18 infusion.

Additional doses of morphine can be given during the ch14.18 infusion to treat neuropathic pain

followed by an increase in the morphine sulfate infusion rate, but patients should be monitored

closely for hypotension and respiratory depression. If patients cannot tolerate morphine (e.g.,

itching), fentanyl or hydromorphone can be substituted for morphine.

e. Alternatively, lidocaine infusion may be used in conjunction with IV bolus of morphine on prn

basis. The administration guidelines for lidocaine infusion are shown below:

Administration of lidocaine:

1. Give lidocaine IV bolus at 2 mg/kg in 50mL NS over 30 minutes prior to the start of the

ch14.18 infusion.

2. At the beginning of the ch14.18 infusion, start IV lidocaine infusion at 1 mg/kg/hour and

continue until two hours after the completion of ch14.18 infusion.

3. May give morphine IV bolus 25-50 microgram/kg every 2 hours prn pain.

4. Patient should be monitored closely for sedation scale, EKG (rate and rhythm), HR, BP,

RR, oxygen saturation (pulse oximeter) and pain score. If patient develops dizziness,

perioral numbness, tinnitus attributable to lidocaine, then stop lidocaine infusion.

f. Gabapentin may also be used in conjunction with IV morphine. Start gabapentin at time of

morphine loading dose, dose gabapentin at 10 mg/kg/day and titrate up to 30-60 mg/kg/day

(maximum dose: 3,600 mg/day) depending on the clinical response.

Have immediately available at the bedside during the ch14.18 infusion:

a. Epinephrine (1:10,000) 0.01 mg/kg or 0.1 mL/kg: IVP (max 5 mL) every 3-5 minutes.

b. Hydrocortisone 2 mg/kg (max 100 mg) IVP. Only use it for life-threatening reactions

(hypotension, bronchospasm, angioedema involving the airway) not responsive to other

measures.

Monitoring during the ch14.18 Infusion:

Vital Signs every 4 hours. During ch14.18 infusion VS every 15 minutes for the first hour

then every 1 hour until completion of antibody infusion, if stable after 1 hour.

Strict I & O every 4 hours, Dipstick urine for heme and SG every void.

Weigh patient twice a day during ch14.18 infusion.

Call Hematology/Oncology MD for:

a. Systolic B/P > 150 or < 70 (>150 or < 65 for infant; >150 or < 80 for patients > 12

years of age) OR a decrease that is more than 15% below baseline; HR > 160 or <

60; Temp > 38.5°C; Respiratory rate > 40.

b. Neuropathic pain requiring an increase in narcotic infusion rate;

c. Urticaria, bronchospasm, peripheral/sensory neurotoxicity, new coughs.

10.2 **International Response Criteria**

Measurable tumor is defined as the product of the longest x widest perpendicular diameter. The third dimension is added when possible. Elevated urine catecholamine metabolite levels and tumor cell invasion of bone marrow also is considered measurable tumor.

10.2.1 Complete Response (CR)

No evidence of primary tumor, no evidence of metastases (chest, abdomen, liver, bone, bone marrow, nodes, etc.), and HVA/VMA normal.

10.2.2 Very Good Partial Response (VGPR)

Greater than 90% reduction of primary tumor; no metastatic tumor (as above except bone); no new bone lesions, all pre-existing lesions improved on bone scan; HVA/VMA normal.

10.2.3 Partial Response (PR)

Fifty-90% reduction of primary tumor; 50% or greater reduction in measurable sites of metastases; 0-1 bone marrow samples with tumor; number of positive bone sites decreased by > 50%.

10.2.4 Mixed Response (MR)

Greater than 50% reduction of any measurable lesion (primary or metastases) with, 50% reduction in any other site; no new lesions; < 25% increase in any existing lesion (exclude bone marrow evaluation).

10.2.5 No Response (NR)

No new lesions; <50% reduction but < 25% increase in any existing lesion (exclude bone marrow

evaluation).

10.2.6 Progressive disease (PD)

Any new lesion or increase of a measurable lesion by > 25%; previous negative or ≤ 10% tumor in marrow increases to > 10% tumor.

7.1 **Required Clinical, Laboratory and Disease Evaluations**

**Obtain prior to start of phase unless otherwise indicated.**

7.1.1Required Observations During the 1st, 3rd and 5th Courses (Ch14.18 + sargramostim (GM-CSF). **Courses #1, 3, 5:** Day 0 is designated as the day of 1st dose of GM-CSF for the 1st course of

immunotherapy


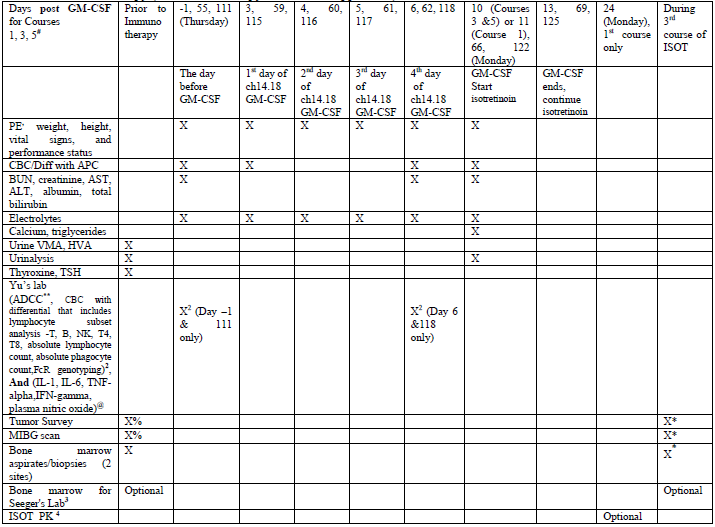


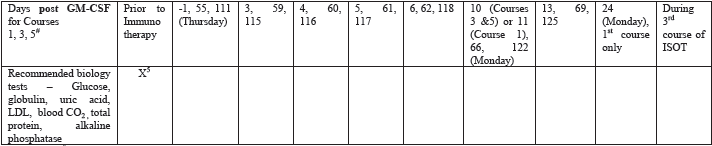


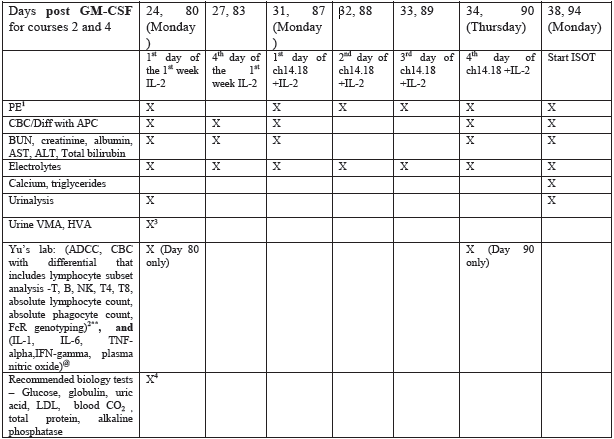


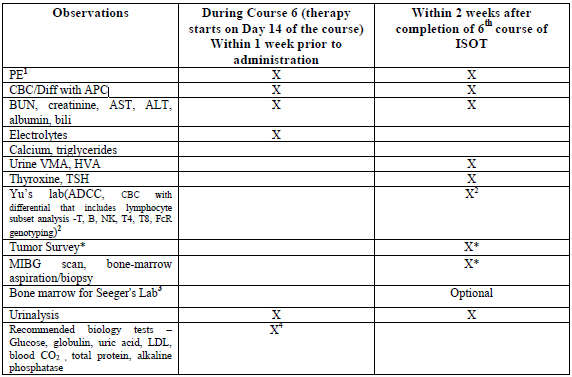


Table 1: Regimens used in patients prior to their enrollment onto COG ANBL0931:

A3973: [Kreissman SG](http://www.ncbi.nlm.nih.gov/pubmed/?term=Kreissman SG%5BAuthor%5D&cauthor=true&cauthor_uid=23890779), [Seeger RC](http://www.ncbi.nlm.nih.gov/pubmed/?term=Seeger RC%5BAuthor%5D&cauthor=true&cauthor_uid=23890779), [Matthay KK](http://www.ncbi.nlm.nih.gov/pubmed/?term=Matthay KK%5BAuthor%5D&cauthor=true&cauthor_uid=23890779), [London WB](http://www.ncbi.nlm.nih.gov/pubmed/?term=London WB%5BAuthor%5D&cauthor=true&cauthor_uid=23890779), [Sposto R](http://www.ncbi.nlm.nih.gov/pubmed/?term=Sposto R%5BAuthor%5D&cauthor=true&cauthor_uid=23890779), [Grupp SA](http://www.ncbi.nlm.nih.gov/pubmed/?term=Grupp SA%5BAuthor%5D&cauthor=true&cauthor_uid=23890779), [Haas-Kogan DA](http://www.ncbi.nlm.nih.gov/pubmed/?term=Haas-Kogan DA%5BAuthor%5D&cauthor=true&cauthor_uid=23890779), [Laquaglia MP](http://www.ncbi.nlm.nih.gov/pubmed/?term=Laquaglia MP%5BAuthor%5D&cauthor=true&cauthor_uid=23890779), [Yu AL](http://www.ncbi.nlm.nih.gov/pubmed/?term=Yu AL%5BAuthor%5D&cauthor=true&cauthor_uid=23890779), [Diller L](http://www.ncbi.nlm.nih.gov/pubmed/?term=Diller L%5BAuthor%5D&cauthor=true&cauthor_uid=23890779), [Buxton A](http://www.ncbi.nlm.nih.gov/pubmed/?term=Buxton A%5BAuthor%5D&cauthor=true&cauthor_uid=23890779), [Park JR](http://www.ncbi.nlm.nih.gov/pubmed/?term=Park JR%5BAuthor%5D&cauthor=true&cauthor_uid=23890779), [Cohn SL](http://www.ncbi.nlm.nih.gov/pubmed/?term=Cohn SL%5BAuthor%5D&cauthor=true&cauthor_uid=23890779), [Maris JM](http://www.ncbi.nlm.nih.gov/pubmed/?term=Maris JM%5BAuthor%5D&cauthor=true&cauthor_uid=23890779), [Reynolds CP](http://www.ncbi.nlm.nih.gov/pubmed/?term=Reynolds CP%5BAuthor%5D&cauthor=true&cauthor_uid=23890779), [Villablanca JG](http://www.ncbi.nlm.nih.gov/pubmed/?term=Villablanca JG%5BAuthor%5D&cauthor=true&cauthor_uid=23890779). Purged versus non-purged peripheral blood stem-cell transplantation for high-risk neuroblastoma (COG A3973): a randomised phase 3 trial. Lancet Oncol. 2013, 14: 999-1008.

ANBL02P1: Park JR, Scott JA, Stewart CF, London WB, Naranjo A, Santana VM, Shaw PJ, Cohn SL, Matthay KK. A pilot induction regimen incorporating pharmacokinetically guided topotecan for treatment of newly diagnosed high risk neuroblastoma: A Children's Oncology Group Study. J Clin Oncol 2011, 33:4351-4357.

NANT2001-02: Gregory A. Yanik, John E. Levine, Katherine K. Matthay, James C. Sisson, Barry L. Shulkin, Brahm Shapiro, David Hubers, Susan Spalding, Tom Braun, James L.M. Ferrara, and Raymond J. Hutchinson Pilot Study of Iodine-131–Metaiodobenzylguanidine in Combination With Myeloablative Chemotherapy and Autologous Stem-Cell Support for the Treatment of Neuroblastoma. J Clin Oncol. 20: 2142-2149, 2002.

ANBL0532 (NCT00567567): Phase III Randomized Trial of Single vs. Tandem Myeloablative Consolidation Therapy for High-Risk Neuroblastoma

ANBL00P1 (NCT00017368): A Pilot Study of Tandem High Dose Chemotherapy with Stem Cell Rescue Following Induction Therapy in Children with High Risk Neuroblastoma (Seif AE, Naranjo A, Baker D, Bunin NJ, Kletzel M, Kretschmar CS, London WB, Maris JM, McGrady PW, Park JR, Diller LR, Grupp SA. A pilot study of tandem high-dose chemotherapy with stem cell rescue as consolidation for high-risk neuroblastoma: Children's Oncology Group study ANBL00P1. Bone Marrow Transplant; 2013 48:947-952).

9640: Granger MP, Grupp SA, Kletzel M, Kretschmar CS, Naranjo A, London WB, Diller L. Feasibility of a tandem autologous peripheral blood stem cell transplant regimen for high risk neuroblastoma in a Cooperative Group Setting: a Pediatric Oncology Group Study: A report from the Children's Oncology Group. Pediatr Blood Cancer; 2012 Nov; 59(5):902-7.

CHP594 or DFCI34-DAT: [George RE](http://www.ncbi.nlm.nih.gov/pubmed/?term=George RE%5BAuthor%5D&cauthor=true&cauthor_uid=16782928)1, [Li S](http://www.ncbi.nlm.nih.gov/pubmed/?term=Li S%5BAuthor%5D&cauthor=true&cauthor_uid=16782928), [Medeiros-Nancarrow C](http://www.ncbi.nlm.nih.gov/pubmed/?term=Medeiros-Nancarrow C%5BAuthor%5D&cauthor=true&cauthor_uid=16782928), [Neuberg D](http://www.ncbi.nlm.nih.gov/pubmed/?term=Neuberg D%5BAuthor%5D&cauthor=true&cauthor_uid=16782928), [Marcus K](http://www.ncbi.nlm.nih.gov/pubmed/?term=Marcus K%5BAuthor%5D&cauthor=true&cauthor_uid=16782928), [Shamberger RC](http://www.ncbi.nlm.nih.gov/pubmed/?term=Shamberger RC%5BAuthor%5D&cauthor=true&cauthor_uid=16782928), [Pulsipher M](http://www.ncbi.nlm.nih.gov/pubmed/?term=Pulsipher M%5BAuthor%5D&cauthor=true&cauthor_uid=16782928), [Grupp SA](http://www.ncbi.nlm.nih.gov/pubmed/?term=Grupp SA%5BAuthor%5D&cauthor=true&cauthor_uid=16782928), [Diller L](http://www.ncbi.nlm.nih.gov/pubmed/?term=Diller L%5BAuthor%5D&cauthor=true&cauthor_uid=16782928). [High-risk neuroblastoma treated with tandem autologous peripheral-blood stem cell-supported transplantation: long-term survival update.](http://www.ncbi.nlm.nih.gov/pubmed/16782928) [J Clin Oncol.](http://www.ncbi.nlm.nih.gov/pubmed) 2006 Jun 20;24(18):2891-6.

Methodological Details of Human Anti-Chimeric Antibody (HACA) Study:

A master mix of B-ch14.18 (biotinylated-ch14.18) and Ru-ch14.18 (ruthenium conjugated ch14.18) was prepared in assay buffer (1% MDS blocker A in 1X PBS containing 0.1% Tween-20), with final concentrations of 0.60 μg/mL Ru-ch14.18 and 0.125 μg/mL of B-ch14.18. Samples were diluted 10-fold (MRD) in assay buffer. Diluted samples (50 μL) were added to 100 μL of master mix in wells of a polypropylene plate. The plate was sealed and incubated for approximately 1 hour at room temperature on a shaker set at approximately 300 rpm. During the sample pre-incubation, an MSD Streptavidin Gold 96-well plate was prepared by adding 150 μL of blocking buffer (5% MSD Blocker A in PBS) to each well. The plate was covered and incubated for approximately 1 hour at room temperature on a shaker set at approximately 300 rpm. Blocking buffer was removed from wells of the MSD Streptavidin Gold plate, and 50 μL of samples, pre-incubated with master mix, was transferred from the polypropylene plate to duplicate wells of the MSD plate. The plate was covered and incubated for approximately 1 hour at room temperature on a shaker set at approximately 300 rpm. The assay plate was washed three times with approximately 300 μL of wash buffer (1X PBS containing 0.05% Tween-20, PBST) per well, and 150 μL of 2X read buffer T (prepared from 4X Read Buffer T) was added to each well. The plate was read on the Sector Imager 6000 (Meso Scale Discovery).
